# Immunoglobulin somatic hypermutation has clinical impact in DLBCL and potential implications for immune checkpoint blockade and neoantigen-based immunotherapies

**DOI:** 10.1186/s40425-019-0730-x

**Published:** 2019-10-22

**Authors:** Zijun Y. Xu-Monette, Jianyong Li, Yi Xia, Beryl Crossley, Robert D. Bremel, Yi Miao, Min Xiao, Thomas Snyder, Ganiraju C. Manyam, Xiaohong Tan, Hongwei Zhang, Carlo Visco, Alexandar Tzankov, Karen Dybkaer, Govind Bhagat, Wayne Tam, Hua You, Eric D. Hsi, J. Han van Krieken, Jooryung Huh, Maurilio Ponzoni, Andrés J. M. Ferreri, Michael B. Møller, Miguel A. Piris, Jane N. Winter, Jeffrey T. Medeiros, Bing Xu, Yong Li, Ilan Kirsch, Ken H. Young

**Affiliations:** 1 Hematopathology Division and Department of Pathology, Duke University School of Medicine, Duke University Medical Center and Cancer Institute,, Durham, NC 27710 USA; 2 Department of Hematopathology, The University of Texas MD Anderson Cancer Center, Houston, TX USA; 3 Adaptive Biotechnologies, Seattle, WA USA; 4 ioGenetics LLC, Madison, WI USA; 5 Department of Bioinformatics and Computational Biology, The University of Texas MD Anderson Cancer Center, Houston, TX USA; 6 San Bortolo Hospital, Vicenza, Italy; 7 Institute of Pathology and Medical Genetics, University Hospital of Basel, Basel, Switzerland; 8 Aalborg University Hospital, Aalborg, Denmark; 9 Columbia University Medical Center and New York Presbyterian Hospital, New York, NY USA; 10 Weill Medical College of Cornell University, New York, NY USA; 11 Affiliated Cancer Hospital & Institute of Guangzhou Medical University, Guangzhou, China; 12 Cleveland Clinic, Cleveland, OH USA; 13 Radboud University Nijmegen Medical Centre, Nijmegen, Netherlands; 14 Asan Medical Center, Ulsan University College of Medicine, Seoul, Korea; 15 San Raffaele H. Scientific Institute, Milan, Italy; 16 Odense University Hospital, Odense, Denmark; 17 Hospital Universitario Marqués de Valdecilla, Santander, Spain; 18 Feinberg School of Medicine, Northwestern University, Chicago, IL USA; 19 Department of Hematology, The First Affiliated Hospital of Xiamen University, Xiamen, China; 20 Department of Medicine, Baylor College of Medicine, Houston, TX USA; 21 Duke University Medical Center and Cancer Institute, Durham, NC 27710 USA

**Keywords:** Immunoglobulin, SHM, Neoantigen, PD-1, MHC, HLA, 9p.24, BCL2, NGS, DLBCL

## Abstract

**Background:**

Diffuse large B-cell lymphoma (DLBCL) harbors somatic hypermutation (SHM) in the immunoglobulin heavy chain and light chain variable region genes, IGHV and IGK/LV. Recent studies have revealed that IGV SHM creates neoantigens that activate T-cell responses against B-cell lymphoma.

**Methods:**

To determine the clinical relevance of IGV SHM in DLBCL treated with standard immunochemotherapy, we performed next-generation sequencing of the immunoglobulin variable regions and complementarity determining region 3 (CDR3) for 378 patients with de novo DLBCL. The prognostic effects of IGV SHM and ongoing SHM or intra-clonal heterogeneity were analyzed in the training (192 patients), validation (186 patients), and overall DLBCL cohorts. To gain mechanistic insight, we analyzed the predicted IG-derived neoantigens’ immunogenicity potential, determined by the major histocompatibility complex-binding affinity and the frequency-of-occurrence of T cell-exposed motifs (TCEMs) in a TCEM repertoire derived from human proteome, microbiome, and pathogen databases. Furthermore, IGV SHM was correlated with molecular characteristics of DLBCL and PD-1/L1 expression in the tumor microenvironment assessed by fluorescent multiplex immunohistochemistry.

**Results:**

SHM was commonly found in IGHV and less frequently in IGK/LV. High levels of clonal IGHV SHM (SHM^high^) were associated with prolonged overall survival in DLBCL patients, particularly those without *BCL2* or *MYC* translocation. In contrast, long heavy chain CDR3 length, the presence of IGHV ongoing SHM in DLBCL, and high clonal IGK/LV SHM in germinal center B-cell–like (GCB)-DLBCL were associated with poor prognosis. These prognostic effects were significant in both the training and validation sets. By prediction, the SHM^high^ groups harbored more potentially immune-stimulatory neoantigens with high binding affinity and rare TCEMs. PD-1/L1 expression in CD8^+^ T cells was significantly lower in IGHV SHM^high^ than in SHM^low^ patients with activated B-cell–like DLBCL, whereas PD-1 expression in CD4^+^ T cells and PD-L1 expression in natural killer cells were higher in IGK/LV SHM^high^ than in SHM^low^ patients with GCB-DLBCL. *PD-L1*/*L2* (9p24.1) amplification was associated with high IGHV SHM and ongoing SHM.

**Conclusions:**

These results show for the first time that IGV SHM^high^ and ongoing SHM have prognostic effects in DLBCL and potential implications for PD-1/PD-L1 blockade and neoantigen-based immunotherapies.

## Background

A characteristic of mature B-cell neoplasms compared with other cancer cells is the somatic hypermutation (SHM) in genes encoding immunoglobulin (IG) heavy chain (IGH) and light chain (kappa or lambda, IGK/L) variable (V) regions. IGV SHM is acquired during antigen-based affinity maturation of activated B cells in the germinal center and mediated by activation-induced cytidine deaminase (AID) [1–4]. AID can also mediate abnormal SHM, abnormal rearrangement of D (diversity), J (joining), and V gene segments (e.g., *BCL2* translocation to the IGHJ region [5, 6]), aberrant class-switch recombination (e.g., *MYC* translocation to the IG switch region) [5–7], and ongoing SHM in malignant B cells, implicated in the pathogenesis and evolution of B-cell neoplasms [2, 8–10].

The prognostic significance of IGV SHM has not been studied in diffuse large B-cell lymphoma (DLBCL), the most common aggressive B-cell lymphoma. In addition to the association with B-cell division and proliferation in the germinal center reaction [3] and abnormal SHM, IGV SHM may enhance the B-cell receptor (BCR) affinity and B-cell survival, suggesting unfavorable prognostic effects. Different from the tonic BCR signaling in germinal-center B-cell–like (GCB)-DLBCL [11, 12], chronic active BCR signaling [13] in activated B-cell–like (ABC)-DLBCL is driven by the self-antigen engagement of BCR and essential for B-cell survival [14]. Self-antigens can be derived from the idiotypic epitope in the BCR’s own V region and engaged with BCR [14].

On the other hand, B-cell IG-derived peptides can be processed and presented to major histocompatibility complex (MHC)-restricted CD4^+^/CD8^+^ T cells [15–18]. In mantle cell lymphoma, somatic neoantigens among all MHC-bound peptides (pMHCs) are exclusively derived from IGV and strongly biased towards MHC-II [18]. These neoantigens are mostly derived from framework region 3 (FW3) and complementarity determining region 3 (CDR3), and are created by either SHM or V-D-J recombination. In contrast, no neoantigenic pMHC were detected for somatically mutated non-IG genes, including *TP53* and *CCND1*, despite the whole-proteomic recovery of non-neoantigenic pMHCs [18]. Similar results were found in follicular lymphoma, DLBCL, and chronic lymphoid leukemia (CLL) [19]. These results suggest that IGV SHM, but not non-IG mutations derived from aging or AID activities, has an important role in shaping the immune response against B-cell lymphomas. However, whether the positive role of IGV-derived neoantigens is significant in patients treated with immunochemotherapy and how the abundance of neoantigens affects the clinical outcome is unknown. A recent study by single-molecule imaging in live primary T cells revealed that with progressively higher pMHC densities, the set point for T-cell receptor (TCR) activation increases, and the cooperativity of pMHC:TCR binding switches from positive to negative [20]. Ii is also known that prolonged antigen exposure under suboptimal costimulatory conditions induces PD-1 expression on T cells which dampens the T-cell response [21].

Our previous in silico analysis found that IG-derived pMHCs’ T-cell exposed motifs (TCEMs), which are important determinants of the cognate interaction with the TCR, are recurrent at a wide range of frequencies in a large IGHV dataset [22]. Some TCEMs were rarely present in the TCEM repertoire built from human proteome, microbiome, and pathogenic bacteria databases [22, 23]. It is logical that T cells encountering abundant high-affinity pMHCs with germline or very common TCEMs remain in a homeostatic balance but mount an active immune response when encountering exogenous or rare TCEMs on high-affinity pMHCs.

In this study, we performed next-generation sequencing (NGS) of the IGV FW3 region and the entire CDR3 and investigated the prognostic significance of IGV SHM and ongoing SHM in 378 DLBCL patients treated with the standard immunochemotherapy regimen. In silico prediction of IG-derived pMHCs, PD-1 and PD-1-ligand 1/2 (PD-L1/2)'s cell-specific expression, *BCL2*/*MYC*/*BCL6* rearrangements and mutations, and BCR signaling biomarkers were analyzed and correlated with SHM to understand the prognostic effects.

## Methods

### Patients

The study cohort is composed of two independent cohorts—a training set and a validation set, sequentially constructed from 21 medical centers in North America and Europe (CONSORT flow diagram in Additional file 1: Figure S1a). Included patients were diagnosed between 1999 and 2009 with de novo DLBCL according to the World Health Organization classification criteria; underwent rituximab, cyclophosphamide, doxorubicin, vincristine, and prednisone (R-CHOP) therapy; and had diagnostic biopsy specimens sufficient for NGS. Patients with transformed DLBCL, primary cutaneous DLBCL, or primary central nervous system DLBCL and HIV-positive patients were excluded. In total, 378 patients (192 training and 186 validation) were sequenced for IGH, and 269 patients also sequenced for IGK/L. The clinical features of the overall, training, and validation cohorts are in Additional file 2: Table S1. By either gene expression profiling (GEP) deposited in GSE#31312 (*n* = 294) or by immunohistochemistry algorithm (*n* = 79) [24, 25], 202 and 171 patients were classified as having GCB-DLBCL and ABC-DLBCL, respectively. Compared with GCB-DLBCL patients, ABC-DLBCL patients had significantly poorer survival (Additional file 1: Figure S1b). This study was part of the International DLBCL Rituximab-CHOP Consortium Program and conducted in accordance with the Declaration of Helsinki [24]. Material transfer agreements were established and approved by the institutional review board of each participating institution, and data collection protocols were approved as being of minimal to no risk or as exempt by the institutional review board of each participating institution.

Of the study cohort, 290 patients having a dominant clonal IG sequence identified were analyzed for prognostic impact. The median age was 63 years, the male-to-female ratio was 1.34, and the median follow-up time was 44.5 months. Molecular characteristics, including B-cell-associated gene signature [26], *BCL2* and *MYC* translocation [27, 28], *MYC* and *BCL6* mutation [29], and various protein expression are available for some patients, with numbers shown in Additional file 1: Figure S2.

### Ultra-deep sequencing

DNA was extracted from formalin-fixed, paraffin-embedded DLBCL specimens using an Invitrogen PureLink genomic DNA kit. DNA samples that passed quantity and quality assessment were subjected to high-throughput immunosequencing of the IGH and IGK/L loci using the immunoSEQ™ platform (Adaptive Biotechnologies, Seattle, WA) [30–32]. An average of 260 ng of genomic DNA was used for each assay; the average sequencing depth of coverage was 162.08x, and the median depth of coverage was 45.57x.

For the IGH locus, a set of multiplexed forward primers matching V (CDR2/FW2) and D gene segment sequences were combined with a set of reverse primers matching J gene segment sequences to amplify both mature V-D-J and immature D-J IGH rearrangements. The reported sequence region by the immunoSEQ hsIGH assay was 130 base pairs starting from the J gene segment. The IGH CDR3 (HCDR3) sequences identified included a fraction of the V region, the complete D and J regions, and random nucleotide insertions. The average sequenced IGHV region was ~ 100 base pairs (including mostly FW3, the CDR3 V fraction, and some CDR2) covering about one-third of the IGHV gene; the median and mean HCDR3 lengths were both 48 base pairs/16 amino acids. For amplifying all possible V-D-J combinations, the assay employed a single-tube, multiplex PCR assay with 84 V and 15 D forward and 9 J reverse primers.

For the removal of potential PCR bias, every possible V-J and D-J pair was chemically synthesized as a template with specific barcodes. These templates were engineered to be recognizable as non-biologic and have universal 3′ and 5′ ends to permit amplification with universal primers and subsequent quantification by high-throughput sequencing. This synthetic immune system could then be used to calibrate the multiplex PCR assay. The multiplex pool of templates was amplified and sequenced iteratively with our IGH V/D- and J-specific primers, and the primer concentrations were adjusted to re-balance PCR amplification. Once the multiplex primer mixture amplified each V and J template nearly equivalently, residual bias was removed computationally.

A similar methodology was used for analyzing the IGK and IGL loci with the immunoSEQ hsIGKL assay, which employed 29 IGK V and 46 IGL V forward primers, plus 6 IGK J and 6 IGL J reverse primers. In addition, kappa deleting element rearrangements with the V region and the intragenic Jκ-Cκ region were also amplified. The reported sequence was ~ 130 base pairs. The median and mean lengths of light chain CDR3 were both 30 base pairs/10 amino acids.

Following high-throughput sequencing, the raw sequencing data were processed with a complexity filter and nearest neighbor algorithm to remove technical failures and correct sequencing errors. A bioinformatics pipeline clustered the sequences into distinct clonotypes based on their CDR3 sequences to determine the overall frequencies of clones. Sequences were delineated according to criteria established by the International ImMunoGeneTics (IMGT) collaboration [33] with a standard algorithm to identify V, D, and J gene segments. Sequences containing premature stop codons or out-of-frame insertions or deletions that resulted in frame shifts were classified as non-productive.

Clones that were relatively expanded with > 5% overall frequency in a sequence repertoire were identified as index trackable sequences. The dominant clones were defined as diagnostic clones representative of the malignant transformation. IGV point mutations were identified by comparing the clonal sequences with the known IMGT germline sequences and assigned as SHM events, allowing a determination of the overall SHM rate. The cutoff for SHM-positive status was > 2% deviation or < 98% identity, as used in CLL routine clinical practice and earlier studies of DLBCL [14, 34, 35].

Intra-clonal IGV variations were further analyzed in SHM-positive cases. Any sequence within the repertoire that included the same point mutations of the same germline sequence as the diagnostic sequence plus at least one additional point mutation was identified as an intra-clonal variant of the diagnostic clone. The cutoff for the presence of ongoing IGHV SHM was ≥2% accumulative frequency of intra-clonal variant sequences in the IGHV repertoire. The cutoff for high IGK/LV ongoing SHM was ≥17 intra-clonal sequence variants.

### MHC-binding prediction

MHC-II binding predictions were made using neural network ensembles (NNEs) trained on MHC II binding data obtained from the IEDB repository (www.iedb.org). We used NNE methods as described previously [36] with the modification that ensembles of neural networks were used. NNE predictions of the Log_e_ of ic_50_ were made for DP (13 genotypes), DQ (28 genotypes), and DR (24 genotypes). All Log_e_ ic_50_ binding predictions were standardized to a common scale for all alleles using a Johnson distribution [37] to transform the raw data into zero mean, unit variance values. The threshold of high-affinity binding was set at − 1 standard deviation from the mean of the zero mean, unit variance values. This approximates the highest 16 percentiles of binding affinity. By way of reference, for the very common DRB01*0101 allele, − 1 standard deviation below the mean converts to an ic_50_ of approximately 50 nM.

Examining the endosomal peptidase cleavage sites indicated that a significant portion of the peptides would be expected to be excised by endosomal cathepsin B, L and S activity [22].

### Frequency-of-occurrence of TCEM

MHC-II TCEMs are derived from one of two discontinuous pentamers of amino acids in the pMHC-II facing outwards and engaging the TCR [22, 38, 39]. A frequency classification (FC) metric was devised to directly index the frequency of cognate T-cell encounters of the particular TCEM, with a log base 2 transformation of the frequency-of-occurrence of 20^5^ TCEM in approximately 50 million immunoglobulin sequences of healthy subjects [23, 40]. The scale of FC ranges from FC1 (high frequency = 1/2^1^) to FC24 (low frequency = 1/2^24^).

### T-cell stimulation metric

For relatively rare TCEMs (FC > 16) in a high-affinity peptide, an empirical stimulation metric was computed using the principle of the additivity of variance across the entire population of allele genes [23]:

$$ Stimulation={\sum}_{a=1}^N{\sigma}_a\ast {2}^{FC-16} $$

Where

$$ a= HLA\  allele, $$

$$ standardized\ binding={\sigma}_a<=-1, $$

and

$$ {-\log}_2\ \mathrm{frequency}=\mathrm{FC}>16 $$

### PD-1/PD-L1/PD-L2 expression and *PDL1*/*L2* genetic analysis

Cell type-specific expression of PD-1 and PD-L1/L2 were quantitated using the fluorescent multiplex immunohistochemistry platform MultiOmyx™; *PDL1*/*L2* copy number alterations were evaluated by fluorescence in situ hybridization as described previously [41]. NGS RNA fusion assay was used to detect *PD-L1*/*2* rearrangement.

### Statistical analysis

Clinical and molecular features were compared using the Fisher exact test and unpaired (2-tailed) t-test. Overall survival (OS) and progression-free survival (PFS) were calculated from the date of diagnosis to the date of last follow-up or death and to the date of disease progression or death, respectively. The survival rates of two groups of patients were compared using Kaplan-Meier curves and the log-rank (Mantel-Cox) test using GraphPad Prism 7. Multivariate analyses with Cox proportional hazards regression models were performed using SPSS statistics 24. *P* values ≤0.05 were considered statistically significant. All comparisons were performed in the overall study cohort and the training and validation sets. The Benjamini-Hochberg procedure was performed for the multiple survival comparisons in the study cohort.

## Results

### High degree of clonal IGHV SHM correlates with favorable prognosis in DLBCL

IGHV index trackable sequences were identified in 224 patients, whereas no clonal sequences showed significant expansion in 65 patients, and the sequencing reads were insufficient for clonal analysis in the other 89 patients. Of the 224 patients with index trackable sequences, 145 had IMGT germline V-D-J sequences identified for diagnostic sequences (Additional file 3), whereas 79 (35%) had only reference D-J sequences resolved in IMGT (CONSORT diagram in Additional file 1: Figure S3).

The distribution of IGHD and IGHV gene usage is shown in Additional file 1: Figure S4a-b. The IGHD3 and IGHV3 families were used most frequently. Consistent with earlier studies [14, 34], IGHV4–34 was significantly overrepresented in ABC-DLBCL compared with GCB-DLBCL (Additional file 1: Figure S4c) but did not have a significant prognostic effect. The distribution of IGHV mutation degree (range, 0–20%) is shown in Additional file 1: Figure S5a; compared with ABC-DLBCL, GCB-DLBCL had a significantly higher mean mutation degree (9.6% vs 7.4%, *P* = 0.012). Most patients (127 of 145, 88%) were SHM-positive. The prognosis of SHM-positive and SHM-negative patients was similar.

**Fig. 1:**
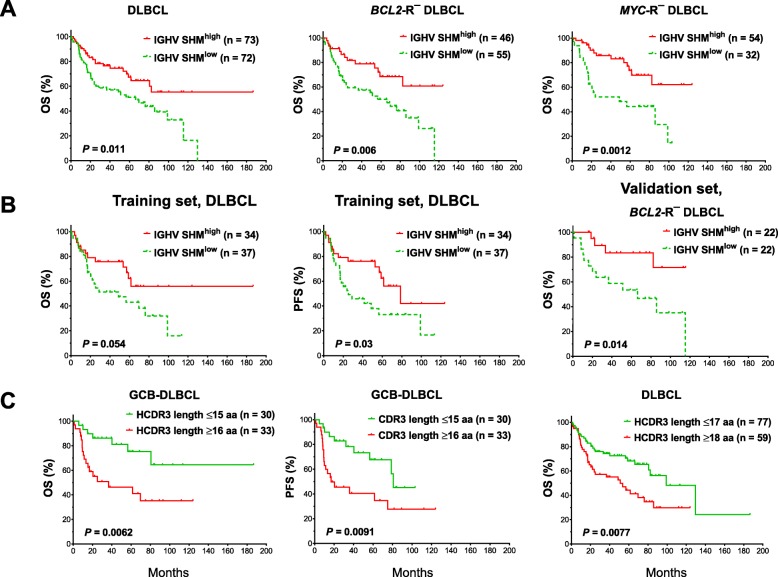
Immunoglobulin heavy chain analysis. **a** A high degree of IGHV SHM (SHM^high^) was associated with significantly better overall survival (OS) in DLBCL overall and in DLBCL lacking *BCL2* rearrangement (*BCL2-*R^−^) or *MYC* rearrangement (*MYC*-R^−^). **b** IGHV SHM^high^ was associated with significantly better OS and progression-free survival (PFS) in the training set, and significantly better OS in the *BCL2-*R^−^ cases of the validation set. **c** Short heavy chain complementarity determining region 3 (HCDR3) length was associated with significantly better OS in the germinal center B-cell-like (GCB)-DLBCL and overall DLBCL

However, with the median SHM degree as the cutoff, SHM^high^ was associated with significantly better OS (*P* = 0.011, Fig. 1a) but not PFS (*P* = 0.10, Additional file 1: Figure S5b). SHM^high^ was associated with a significantly higher frequency of *BCL2* (but not *MYC*) translocation (*BCL2*-R) in DLBCL overall (28.1%, Table 1) and in GCB-DLBCL (55%) (Additional file 2: Table S2), which may have confounded the prognostic analysis. After the exclusion of patients with *BCL2*-R^+^ DLBCL, SHM^high^ was associated with significantly better OS (*P* = 0.006, Fig. 1a) and PFS (*P* = 0.012) in *BCL2*-R^−^ patients. Similar favorable effects of SHM^high^ were found in *MYC*-R^−^ patients (for OS, *P* = 0.0012, Fig. 1a; for PFS, *P* = 0.0047). When partitioning DLBCL into GCB and ABC subtypes, the favorable prognostic effect of IGHV SHM^high^ was significant in ABC-DLBCL and marginally significant in *BCL2*-R^−^ and *MYC*-R^−^ GCB-DLBCL (for OS, *P* = 0.059 and 0.066, respectively; Additional file 1: Figure S5c-d). Multivariate analysis with adjustment for clinical factors (Additional file 2: Table S2–S3) and *MYC*-R revealed that IGHV-SHM^high^ was an independent prognostic factor for significantly longer PFS in patients with ABC-DLBCL (Additional file 2: Table S4).

**Table 1:** Clinicopathologic and molecular characteristics of patients with DLBCL with a low or high degree of SHM in immunoglobulin variable region genes

	IGHV SHM^low^	IGHV SHM^high^	*P*	IGK/LV SHM^low^	IGK/LV SHM^high^	*P*
	n (%)	n (%)		n (%)	n (%)	
Age						
< 60 years	32 (44%)	25 (36%)	.31	66 (40%)	14 (37%)	.72
≥ 60 years	40 (56%)	45 (64%)		97 (60%)	24 (63%)	
Sex						
Male	35 (49%)	41 (59%)	.24	94 (58%)	26 (68%)	.27
Female	37 (51%)	29 (41%)		69 (42%)	12 (32%)	
Stage						
I - II	29 (43%)	34 (52%)	.39	72 (46%)	14 (39%)	.58
III - IV	39 (57%)	32 (48%)		86 (54%)	22 (61%)	
B symptoms						
No	38 (56%)	43 (63%)	.48	103 (66%)	28 (78%)	.23
Yes	30 (44%)	25 (37%)		53 (34%)	8 (22%)	
Serum LDH level						
Normal	21 (33%)	29 (45%)	.20	67 (46%)	18 (55%)	.44
Elevated	43 (67%)	35 (55%)		78 (54%)	15 (45%)	
No. of extranodal sites						
0 or 1	51 (76%)	53 (82%)	.53	126 (81%)	26 (70%)	.18
≥ 2	16 (24%)	12 (18%)		30 (19%)	11 (30%)	
ECOG performance status						
0 or 1	47 (80%)	44 (76%)	.66	113 (79%)	26 (81%)	1.0
≥ 2	12 (20%)	14 (24%)		30 (21%)	6 (19%)	
Largest tumor size						
< 5 cm	37 (66%)	27 (53%)	.17	73 (57%)	13 (43%)	.22
≥ 5 cm	19 (34%)	24 (47%)		55 (43%)	17 (57%)	
IPI score						
0–2	40 (60%)	42 (64%)	.72	97 (62%)	21 (57%)	.58
3–5	27 (40%)	24 (36%)		60 (38%)	16 (43%)	
Therapy response						
CR	51 (71%)	54 (77%)	.45*	126 (77%)	27 (73%)	.67*
PR	10	8		21	3	
SD	2	3		4	1	
PD	9	5		12	6	
ABC subtype/ABC subtype						
GCB	27 (39%)	39 (53%)	.094	78 (47%)	21 (57%)	.36
ABC	43 (61%)	34 (47%)		88 (53%)	16 (43%)	
BAGS classification						
CC/CB	24 (52%)	40 (75%)	**.02**	77 (64%)	21 (81%)	.11
Others	22 (48%)	13 (25%)		43 (36%)	5 (19%)	
*BCL2* translocation						
No	55 (87%)	46 (72%)	**.047**	125 (81%)	22 (65%)	**.042**
Yes	8 (13%)	18 (28%)		29 (19%)	12 (35%)	
MYC expression						
< 70%	39 (57%)	50 (72%)	**.047**	117 (72%)	26 (68%)	.69
≥ 70%	29 (43%)	19 (28%)		46 (28%)	12 (32%)	
PI3K expression						
< 70%	43 (67%)	50 (75%)	.44	112 (72%)	14 (42%)	**.0018**
≥ 70%	21 (33%)	17 (25%)		43 (28%)	19 (58%)	
p63 expression						
< 10%	47 (68%)	38 (54%)	.086	93 (59%)	14 (39%)	**.04**
≥ 10%	22 (32%)	33 (46%)		65 (41%)	22 (61%)	
CD30 expression						
< 20%	58 (83%)	60 (83%)	1.0	135 (82%)	37 (97%)	**.012**
≥ 20%	12 (17%)	12 (17%)		30 (18%)	1 (3%)	
p65 expression						
< 10%	22 (35%)	29 (43%)	.47	61 (41%)	7 (21%)	**.031**
≥ 10%	40 (65%)	39 (57%)		89 (59%)	27 (79%)	
CXCR4 expression						
< 20%	43 (73%)	47 (70%)	.84	109 (72%)	19 (53%)	**.029**
≥ 20%	16 (27%)	20 (30%)		42 (28%)	17 (47%)	

**Abbreviations**: *IGHV* Immunoglobulin heavy chain variable region gene, *IGK/LV* Immunoglobulin kappa or lambda light chain variable region gene, *SHM*^*low*^ Low degree of somatic hypermutation, *SHM*^*high*^ High degree of somatic hypermutation, *LDH* Lactate dehydrogenase, ECOG Eastern Cooperative Oncology Group, *IPI* International Prognostic Index, *CR* Complete response, *PR* Partial response, *SD* Stable disease, *PD* Progressive disease, *GCB* germinal center B-cell–like, *ABC* Activated B-cell–like, *BAGS* B-*cell*–associated gene signature, *CB* Centroblast subtype, *CC* Centrocyte subtype

**Note:** Not all patients had data available. Significant *P* values (Fisher’s exact test) are in bold. *For therapy response, *P* values were for comparisons between CR and non-CR cases

When examining in the training and validation sets separately, in the training set, IGHV SHM^high^ was associated with better OS and PFS with and without the exclusion of patients with *BCL2*-R^+^ DLBCL; in the validation set, IGHV SHM^high^ was associated with significantly better OS only after the exclusion of patients with *BCL2*-R^+^ DLBCL (Fig. 1b). Together, these results confirmed the favorable effects of IGHV SHM^high^ in DLBCL, although the significance may differ in DLBCL subsets.

### Shorter HCDR3 length correlates with favorable prognosis in DLBCL

V-D-J resolved diagnostic sequences were rarely unproductive; only 7 patients had nonsense or out-of-frame mutations. GCB-DLBCL patients with a shorter (< median/mean) amino acid length of HCDR3 (hypervariable sequences) had significantly better OS (*P* = 0.0062) and PFS (*P* = 0.0091; Fig. 1c) despite having a significantly higher proportion of stage III/IV disease (Additional file 2: Table S5). With a cutoff of 2 amino acids higher than the median/mean, short length was associated with significantly better OS (*P* = 0.0077; Fig. 1c) and PFS (*P* = 0.002) in overall DLBCL and showed a trend towards better PFS in ABC-DLBCL (*P* = 0.054; Additional file 1: Figure S6a). In multivariate analysis, short HCDR3 length was a favorable prognostic factor independent of clinical parameters in only GCB-DLBCL (Additional file 2: Table S4). In line with earlier findings that CDR3 shortening is associated with SHM [42], shorter HCDR3 length was associated with higher mean IGHV SHM in GCB-DLBCL, and higher IGK/LV SHM in ABC-DLBCL (Additional file 1: Figure S6b).

In both the training and validation sets, the favorable prognostic effects of short HCDR3 length were significant. The effects in ABC- and GCB-DLBCL were significant in the training and validation set, respectively (Additional file 1: Figure S6c-d).

### IGHV SHM^high^ is associated with increased predicted neoantigens with rare neoepitopes and lower PD-1 expression in CD8 T cells in ABC-DLBCL

**Fig. 2:**
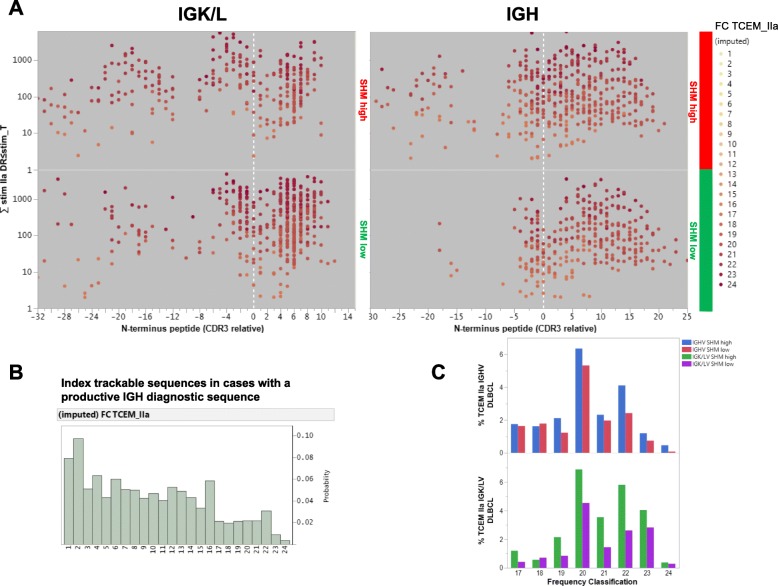
Predicted MHC-binding peptides for immunoglobulin diagnostic sequences and frequency of T-cell exposed motifs (TCEMs). **a** Regional distribution of relatively rare neoantigens (TCEM frequency classification [FC]> > 16) derived from light chain (left) and heavy chain (right) immunoglobulin genes in DLBCL patients. Protein sequences are aligned with cysteine at the start of complementarity determining region 3 (CDR3) at the 0 of the X axis; peptides upstream of CDR3 were defined as framework region 3 (FW3). The stimulation metric was computed using the principle of the additivity of variance and is a product of the standardized MHC-II-binding affinity multiplied by the FC summed over all HLA-DR alleles. Each dot represents one peptide predicted as having high MHC-II-binding affinity (exceeding the − 1 standard deviation threshold for MHC derived from 24 HLA-DR alleles) and relatively rare TCEMs (FC > 16). The color intensities of the dots are scaled on the FC scale, which ranges from FC16 to the very rare FC24. **b** Histograms showing the distribution of the FC of the TCEMs in all MHC-II-binding peptides predicted for index trackable sequences. The FC scale ranges from the commonly presented FC1 to the very rare FC24. **c** Compared with cases without a high degree of heavy chain or light chain IGV SHM, cases with high degree of heavy chain or light chain IGV SHM had higher frequencies of relatively rare TCEMs (FC > 16)

Consistent with earlier studies [18, 19], large numbers of IG-derived peptides were predicted to bind MHC-II (but not MHC-I) with high affinity in patients with a productive IGH diagnostic sequence. The IGHV-SHM^high^ group Compared with the IGHV-SHM^low^ group had significantly more peptides with high HLA-DR-binding affinity predicted (3027 vs. 2688, ~ 16% of total peptides), with either germline (FC < 10, frequency > 1/2^10^) or mutated TCEMs. The stimulation metric for TCEMs with an FC > 16 (relatively rare neoepitopes), which are potentially immune reactive, are plotted in Fig. 2a. These neoepitopes were a minority among patients’ TCEM repertoire identified from all index trackable sequences, as shown by the FC histogram (Fig. 2b). Compared with the IGHV-SHM^low^ group, the IGHV-SHM^high^ group had more pMHCs with TCEM FC > 16 derived from the CDR3 (303 vs. 258) and FW3 (140 vs. 65) regions, an increased percentage of FW3 origin (4.6% vs 2.4%), and an increased percentage of rare TCEMs with an FC of 19–24 (more rare neoepitopes; Fig. 2c). A similar pattern of differences in pMHCs and neoepitopes between the SHM^high^ and SHM^low^ groups was found in the *BCL2*-R^−^, *MYC*-R^−^, and ABC-DLBCL subcohorts as well as the training and validation sets (Additional file 1: Figure S7a-b).

**Fig. 3:**
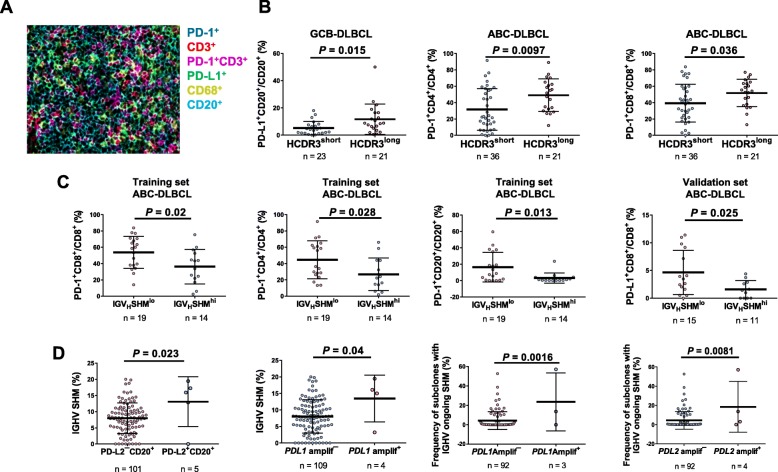
Comparison of PD-1 expression between groups. **a** A representative image of a DLBCL sample is from an ABC-DLBCL case with a low degree of IGHV SHM (2.94%) and a long (21 amino acids) heavy chain complementarity determining region 3 (HCDR3). Fluorescence multiplex immunohistochemistry detected that PD-1 was expressed in T cells and proximal to PD-L1-expressing B cells. **b** Long HCDR3 length was associated with high PD-L1 expression in B cells in GCB-DLBCL and high PD-1 expression in CD4^+^/CD8^+^ T cells in ABC-DLBCL. **c** In the training set, a high degree of IGHV SHM (SHM^hi^) was associated with low PD-1 expression in CD8^+^/CD4^+^ T cells and B cells in ABC-DLBCL. In the validation set, IGHV SHM^hi^ was associated with lower PD-L1 expression in CD8^+^ T cells. **d** PD-L2 protein expression in B cells was associated with a high degree of IGHV SHM. *PD-L1* gene amplification was associated with a significantly higher mean degree of SHM in the IGHV diagnostic sequence. *PD-L1/L2* gene amplification was associated with a higher mean percentage of subclones with IGHV ongoing SHM in the sequence repertoire

To gain insight into the immune surveillance in the tumor microenvironment, fluorescent mIHC was performed to evaluate immune cell-infiltration and cell-specific PD-1/L1/L2 expression (representative image in Fig. 3a) [41], correlating with IGHV SHM and CDR3 length. Long HCDR3 length was associated with higher PD-L1 expression in B cells in GCB-DLBCL (Fig. 3b; significant in the training set; marginally significant in the validation set) and higher PD-1 expression in CD4^+^/CD8^+^ T cells in ABC-DLBCL (Fig. 3b; significant in the validation set; strong trends in the training set). In ABC-DLBCL, IGHV-SHM^high^ was associated with significantly lower PD-1 expression in T cells and B cells in the overall cohort and the training set, and significantly lower PD-L1 expression in CD8^+^ T cells in the overall cohort and the validation set (Fig. 3c). In the overall ABC-DLBCL cohort, IGHV SHM^high^ cases compared with SHM^low^ cases had significantly lower mean cellularity of CD4^+^ T cells but similar cellularity of CD8^+^ T cells (Additional file 1: Figure S7c). B-cell PD-L2 expression and *PD-L1*/*PD-L2* gene amplification (very low frequency in the study cohort, predominantly found in ABC-DLBCL) were associated with high IGHV SHM (Fig. 3d).

Together, these findings suggest that the IGHV-SHM^high^ group produced more T-cell stimulatory neoantigens, which may be relevant for PD-1 expression regulation and function of cognate T cells.

### Ongoing IGHV SHM correlates with significantly poorer survival in DLBCL

**Fig. 4:**
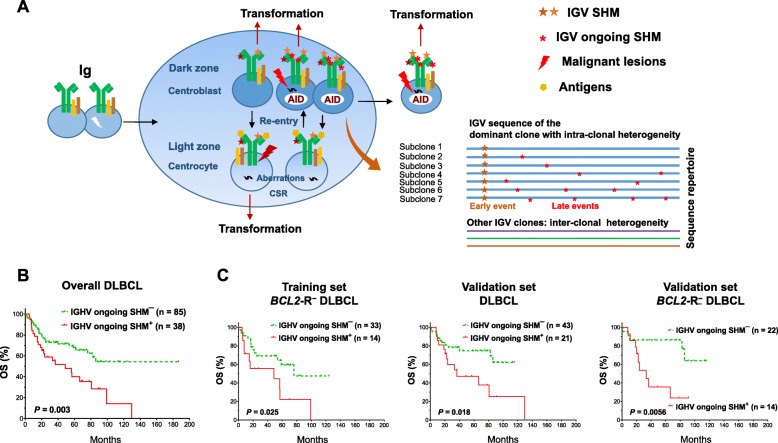
Prognostic analysis for IGHV ongoing SHM. **a** Schematic illustration of the putative pathologic origins of IGV SHM and ongoing SHM in DLBCL founder clones and subclones. Transformation can occur in different stages of B-cell development. When DLBCL abnormalities are sufficient to drive lymphomagenesis, DLBCL cells exit the germinal center reaction. Predominant DLBCL clones may exhibit intra-clonal IGV variations conferred by the ongoing SHM process. **b** IGHV ongoing SHM was associated with significantly poorer overall survival (OS) in the overall study cohort. **c** IGHV ongoing SHM was associated with poorer OS in the overall validation cohort and in cases without *BCL2* rearrangement (*BCL2-*R^−^) in both the training and validation sets

Intra-clonal sequence variations (Fig. 4a) were identified in 102 (83%) of the productive IGHV SHM-positive cases (most frequently in the IGHV3 and IGHV4 families; Additional file 1: Figure S8a). With a cutoff of subclonal frequency at the 70th percentile, ongoing IGHV SHM was associated with significantly poorer OS in patients with DLBCL in the univariate analysis (*P* = 0.003; Fig. 4b) and poorer OS and PFS in the multivariate analysis (Additional file 2: Table S4). The adverse prognostic effect was significant regardless of GCB/ABC and *MYC-*R status and was significant in *BCL2*-R^−^ (for OS, *P* = 0.007, for PFS, *P* = 0.01) but not *BCL2*-R^+^ patients. Similar prognostic results were found in both the training and validation cohorts (Fig. 4c).

Ongoing IGHV SHM was associated with *AICDA* upregulation in overall DLBCL and the validation set. *PD-L1*/*PD-L2* gene amplification and macrophage PD-L2 expression were associated with higher ongoing SHM (Fig. 3d, Additional file 1: Figure S8b).

### IGK/LV SHM^high^ correlates with significantly poorer survival in patients with GCB-DLBCL

Light chain diagnostic sequences were identified in 205 (76%) DLBCL patients (CONSORT diagram in Additional file 1: Figure S3). Consistent with the order of rearrangement, IGL clones were seen only in patients with unproductive IGK. No prognostic difference was observed between the kappa and lambda types. Compared with IGHV, IGK/LV had significantly fewer mutations. The frequency of IGK/LV SHM-positive cases was 53.6% (105 of 205). There were many more IGK clones with no SHM than IGH or IGL clones with no SHM (Additional file 1: Figure S8c). IGLV-SHM had higher correlation with IGHV-SHM than IGKV-SHM (Additional file 1: Figure S8d).

**Fig. 5:**
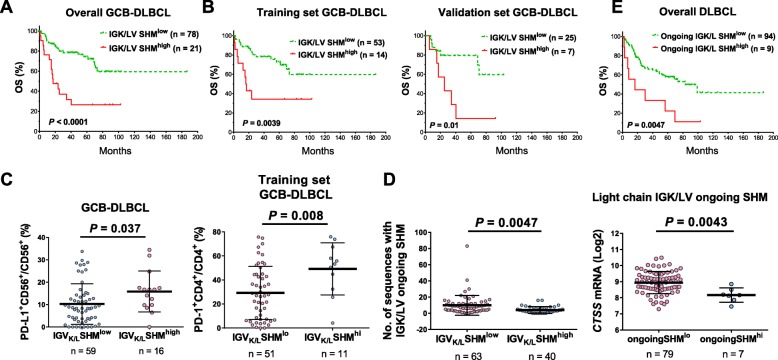
Prognostic and correlative analyses for light chain IGK/LV SHM. **a** A high degree of IGK/LV SHM (SHM^high^) was associated with significantly worse overall survival (OS) in GCB-DLBCL. **b** The adverse prognostic effect of IGK/LV SHM^high^ in GCB-DLBCL was significant in both the training and validation sets. **c** IGK/LV SHM^high^ was associated with higher PD-L1 expression in CD56^+^ natural killer cells in overall GCB-DLBCL cases and with high PD-1 expression in CD4^+^ T cells in the training set. **d** There was a negative correlation between light chain IGK/LV ongoing SHM and IGK/LV SHM. High IGK/LV ongoing SHM was associated with low *CTSS* mRNA expression. **e** High numbers (≥17) of subclones with IGK/LV ongoing SHM were associated with significantly poorer OS in DLBCL

IGK/LV SHM-positive status was not associated with prognostic effect. However, with a high cutoff close to the 80th percentile, IGK/LV SHM^high^ was associated with significantly poorer OS and PFS in patients with GCB-DLBCL (*P* < 0.0001 for OS, Fig. 5a; *P* = 0.0016 for PFS); the effects were confirmed in both the training and validation cohorts (Fig. 5b, Additional file 1: Figure S9a) and by multivariate analysis (Additional file 2: Table S4). Like IGHV SHM^high^, IGK/LV SHM^high^ was associated with a higher frequency of *BCL2*-R in DLBCL (35%, Table 1). However, the adverse prognostic effect of IGK/LV SHM^high^ was independent of *BCL2*-R and *MYC*-R status and was strongest in *BCL2*-R^+^ GCB-DLBCL (Additional file 1: Figure S9b-c).

A short K/LCDR3 length (≤12 aa) was associated with significantly better OS in DLBCL overall and in ABC-DLBCL (*P* = 0.026 and 0.012, respectively; Additional file 1: Figure S9d). However, the prognostic effect was only significant in the validation set (*P* = 0.015; it showed a nonsignificant trend in the training set of ABC-DLBCL, *P* = 0.15), and the number of cases with long K/LCDR3 length was small (4 and 3 in the training and validation sets, respectively).

### IGK/LV SHM^high^ is associated with increased rare neoepitopes and PD-1 expression on CD4^+^ T cells in GCB-DLBCL

The T-cell stimulation metric for predicted MHC-II neoantigens derived from productive IGK/L diagnostic sequences is shown in Fig. 2a. Because the IGK/L SHM^high^ and SHM^low^ groups had unbalanced numbers of patients, the groups’ mean numbers of predicted pMHC-II were compared. IGK/LV SHM^high^ patients had a larger mean number (8.4 vs 4.5 per patient) and percentage (FW3-origin, 10% vs 2.7%; CDR3-origin, 9.1% vs 7.2%) of predicted pMHC-II with FC > 16 TCEMs, but not total predicted pMHC-II (44 vs 46 per patient). The association of IGK/L SHM^high^ with more pMHC-II with FC > 16 TCEMs per patient was observed in both the training and validation sets.

Compared with IGK/LV SHM^low^ patients, IGK/LV SHM^high^ patients had significantly higher PD-L1 expression in natural killer cells (*P* = 0.037; Fig. 5c) and higher *CTSL1* (lysosomal protease genes cathepsin L [43]) mRNA expression in GCB-LDBCL (*P* = 0.038; Additional file 1: Figure S9e), but significantly lower B-cell PD-1 expression (*P* = 0.03) in ABC-DLBCL (Additional file 1: Figure S9f). In contrast, IGHV SHM^high^ was associated with lower *CTSF* expression in GCB-DLBCL (*P* = 0.048; Additional file 1: Figure S9e). In the training but not the validation set, IGK/LV SHM^high^ patients had higher PD-1 expression in CD4^+^ T cells in GCB-DLBCL (*P* = 0.008, Fig. 5c) and higher *AICDA* mRNA in ABC-DLBCL (*P* = 0.047).

Because the correlation findings were differential in the training/validation sets and in the GCB/ABC subtypes, these subsets/subtypes were compared. Compared with the validation set, the training set had significantly higher mean mRNA levels of several MHC-II genes (*HLA-DPA1*, *HLA-DPB1*, *HLA-DRA*, *HLA-DRB1*/*4*) and lysosomal protease genes (*CTSH*, *ASNS*, and *GILT*) (expression data were extracted from the GEP #31312 deposit; Additional file 1: Figure S10a). These differences were largely attributable to the validation set’s *MYC*-R^+^ cases (Additional file 1: Figure S10b), and there were no significant expression differences (except for *CTSH*) between validation set’s *MYC*-R^−^ cases and the training set. In both the training and validation sets, *MYC*-R was associated with downregulation of *HLA-F*, *CTSH*, and *CTSK* in DLBCL and GCB-DLBCL.

In both the training and validation sets, ABC-DLBCL compared with GCB-DLBCL had higher macrophage and CD8^+^ T-cell infiltration, higher PD-L1^+^ expression in B cells (Additional file 1: Figure S10c for the overall cohort), higher *HLA-C*/*E*, *CTSZ*, and *CTSC* mRNA, and lower *HLA-DQB2*, *HLA-DRB4*, and *CTSK* mRNA expression. In the training set only, ABC compared with GCB subtype had significantly higher *CTSB*, *CTSL1*, and *CTSS* expression, and in the validation set only, significantly higher *CTSL3* expression and lower *CTSF* Expression.

### High intra-clonal IGK/LV diversity is associated with unfavorable prognosis

Of the 103 productive IGK/LV SHM-positive cases, 91 (88%) had intra-clonal IGK/L variants (ongoing SHM). The numbers of sequences with ongoing IGK/LV SHM showed negative association with IGV SHM (Fig. 5d, Additional file 1: Figure S11a) and *CTSS* (a cathepsin with an essential role in proteolytic processing of MHC class II-associated invariant-chain peoptide fragments [43]) mRNA levels (Fig. 5d). *PD-L1* polyploidy, exclusively found in GCB-DLBCL, was associated with ongoing IGK/LV SHM (Additional file 2: Table S6).

High intra-clonal IGK/L diversity (≥17 subclones), present in only 9 patients (8 were GCB-DLBCL), was associated with unfavorable clinical parameters, significantly poorer OS/PFS, and distinct gene signatures in DLBCL and GCB-DLBCL (Fig. 5e, Additional file 1: Figure S11b-c, Additional file 2: Table S6–S7). However, the prognostic effects were significant only in the training set (Additional file 1: Figure S11d) and not significant in the multivariate analysis.

**Fig. 6:**
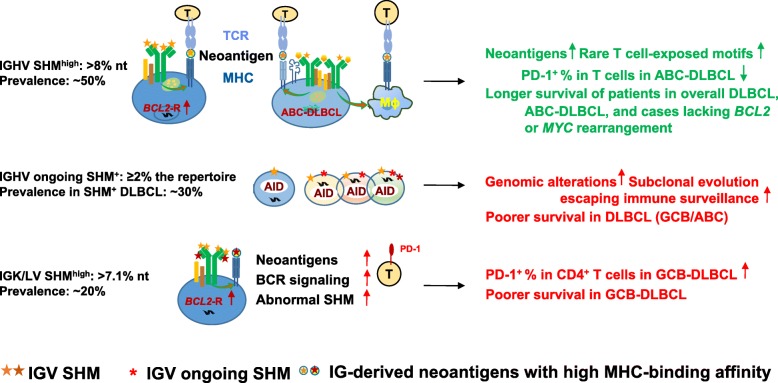
Schematic summary of the prognostic effects of IGV clonal SHM and ongoing SHM in DLBCL and putative underlying mechanisms suggested by in silico analysis and fluorescent multiplex immunohistochemistry and conventional chromogenic immunohistochemistry experiments. Abbreviations: Ig, immunoglobulin protein; AID, activation-induced cytidine deaminase; CSR, class-switch recombination; TCR, T-cell receptor; MHC, major histocompatibility complex; BCR, B-cell receptor; Mɸ, macrophage

Multiple comparison correction was performed (Additional file 2: Table S8) and the validated prognostic effects with potential underlying mechanisms are illustrated in Fig. 6.

## Discussion

IGV SHM, which is distinguished from scattered genome-wide aging-associated non-IG somatic mutations by high mutation density and protein expression [44], has an essential role in neoantigen presentation [18, 44]. However, the clinical relevance of IGV SHM is less studied than that of non-IG mutations, likely owing to technical and interpretive difficulties. In this study, IGV SHM^high^ and ongoing SHM identified through NGS showed prognostic significance in a large cohort of patients with de novo DLBCL treated with R-CHOP, which was validated in the training and validation sets.

First, IGHV SHM^high^ was associated with significantly longer OS in DLBCL patients and longer OS and PFS in DLBCL patients without *MYC*/*BCL2* translocations, which is reminiscent of the favorable PFS and OS incrementally associated with IGHV% deviation in CLL patients [45]. Consistent with the favorable prognostic effect, IGHV-SHM^high^ patients had more enriched MHC-II neoantigens with rare neoepitopes by in silico prediction [22] but lower T-cell PD-1 expression in ABC-DLBCL. The implications of IGHV SHM for T-cell response activation and regulation warrants future study for functional validation and therapeutic exploration. A study showed that treatment with CpG, a TLR9 agonist, promoted MHC-II presentation of IG-derived neoantigens of mantle cell lymphoma cells [19].

Second, compared with IGHV, IGK/LV had less SHM, but IGK/LV SHM^high^ was associated with significantly poorer OS and PFS and high PD-1 expression in CD4^+^ T cells and PD-L1 in natural killer cells in GCB-DLBCL, even though FW3-derived MHC-II neoantigens with rare neoepitopes were significantly higher in IGK/LV SHM^high^ DLBCL compared with IGHV SHM^high^ DLBCL (4.4 vs 2 per patient) and IGH/K/LV SHM^low^ DLBCL (1 per patient). These results appeared to suggest that the excessive neoantigens in IGK/LV SHM^high^ patients with GCB-DLBCL had a negative role in T-cell response by inducing PD-1. In addition, IGK/LV SHM^high^ in GCB-DLBCL could be a biomarker for stronger BCR affinity and higher B-cell proliferation propensity [3, 14], therefore synergizing with unfavorable *BCL2*-R which enhanced cell survival. This is supported by the mutually exclusive pattern of IGK/LV SHM^high^ and IGK/LV ongoing SHM^high^, suggesting a survival advantage of the expanded IGK/LV-SHM^high^ clone, leading to intra-clonal homogeneity.

Third, the presence of IGHV ongoing SHM or intra-clonal heterogeneity had an adverse prognostic effect in SHM-positive patients. Whether the adverse prognosis resulted from subclonal evolution, such as the selection of clones with less immunogenicity [46], loss of MHC expression, or enhanced cell survival, could be revealed by collecting serial tumor biopsy specimens during and after therapy in future prospective studies and subjecting them to longitudinal NGS and flow cytometry experiments to monitor the clonal evolution. The higher ongoing SHM in DLBCL patients than in CLL patients and its adverse prognostic effect in IGHV SHM-positive case, may explain why SHM-positivity status lacks a favorable prognostic effect in DLBCL but not CLL [45, 47].

In addition, as chromosome 9p24.1 amplification has been correlated with the efficacy of PD-1 blockade in Hodgkin lymphoma [48], it would be interesting to investigate the biomarker value of IGHV SHM^high^ and IGV ongoing SHM for clinical response to PD-1 blockade immunotherapy in DLBCL, which showed associations with 9p24.1 amplification and PD-1 expression in the current study. In melanoma patients treated with anti-PD-1 immunotherapy, high tumor clonal mutation load was associated with improved overall survival and higher TCR-clonality (less diverse repertoire) predicted response to anti-PD-1 immunotherapy [49, 50].

## Conclusions

In summary, clonal IGHV SHM^high^ had favorable prognostic effect in patients with DLBCL without *BCL2*/*MYC* translocation, whereas IGHV ongoing SHM and clonal IGK/LV SHM^high^ had adverse prognostic effects in DLBCL and GCB-DLBCL patients, respectively. Neoantigen loads, PD-1/PD-L1 immune checkpoint, and BCR affinity and signaling may contribute to these prognostic effects. IGV SHM evaluation has implications for the selection of PD-1/PD-L1 inhibitors, BCR-targeted agents, and effective vaccines in DLBCL patients. Because NGS is available in clinical practice, the application of IG NGS with immunoSEQ is feasible and can improve risk stratification at diagnosis and identification of dominant tumor clones in lymphoma. Future studies are warranted to determine the value of IG NGS in tracking resistant clones expanded at relapse and in indicating response to immunotherapy and to investigate the therapeutic potential of IG-based vaccines and how IG-derived neoantigens shape the immune response.

## Supplementary information

**Additional file 1: Fig S1.**. Construction and clinical outcome of the diffuse large B-cell lymphoma (DLBCL) cohort. **Fig. S2.** Diagram showing numbers of cases in this mutation study that have been characterized by various biomarker studies, and survival rates of patients whose sequencing results were correlated with prognosis. **Fig. S3.** CONSORT flow diagram illustrating the number of cases performed for high-throughput IG sequencing and clonal sequence analysis. **Fig. S4.** Molecular characterization for immunoglobulin heavy chain (IGH) gene usage in the study cohort. **Fig. S5.** Immunoglobulin heavy chain V gene (IGHV) somatic hypermutation (SHM) analysis. **Fig. S6.** Analysis for length of heavy chain CDR3. **Fig. S7.** Prediction of MHC-binding peptides and frequency of T-cell exposed motifs (TCEM) for immunoglobulin diagnostic sequences in the training set and validation set. (a) Regional distribution of relatively rare neoantigens derived from heavy chain and light chain immunoglobulin genes in DLBCL patients in the training set (top) and validation set (bottom). (b) Cases with high degree of heavy chain or light chain IGV SHM compared with cases without had higher frequency of relatively rare TCEM in the training (left) and validation sets (right). (c) In ABC-DLBCL, high IGV SHM was associated with lower tissue cellularity of CD4+ T cells. **Fig. S8.** Moleclar analysis for immunoglobulin heavy chain ongoing SHM and light chain SHM. **Fig. S9.** Immunoglobulin light chain SHM and CDR3 analysis. **Fig S10.** Comparison between different subsets of DLBCL. **Fig S11.** Light chain IGK/LV ongoing SHM analysis.

**Additional file 2: Table S1.** Clinical features of 378 patients in the training and validation cohort whose DLBCL biopsies were sequenced and 290 patients whose sequencing results showed sufficient sequence reads. **Table S2.** Comparisons of clinicopathologic and molecular characteristics between patients with germinal-center B-cell–like (GCB) DLBCL with a low or high degree of somatic hypermutation (SHM) in immunoglobulin variable region genes. **Table S3.** Comparisons of clinicopathologic and molecular characteristics between patients with activated B-cell-like (ABC) subtype of DLBCL with a low or high degree of SHM in immunoglobulin variable region genes. **Table S4.** Significant prognostic effects of immunoglobulin molecular characteristics in DLBCL patients treated with R-CHOP by multivariate survival analysis. **Table S5.** Clinicopathologic and molecular characteristics of patients with DLBCL with a short or long immunoglobulin heavy/light chain CDR3 length. **Table S6.** Clinicopathologic and molecular characteristics of patients with DLBCL with ongoing SHM in immunoglobulin variable region genes. **Table S7.** Gene signatures associated with SHM in immunoglobulin sequences of DLBCL samples. **Table S8.** Multiple testing corrections for prognostic effects found in the overall cohort of DLBCL treated with R-CHOP by the Benjamini-Hochberg method with a false discovery rate of 0.10

**Additional file 3.** Diagnostic immunoglobulin heavy chain gene sequences

## Data Availability

The datasets used and/or analyzed during the current study are available from the corresponding author on reasonable request based on the condition that IRB and MTA could be approved from the institutions.

## Abbreviations

ABC: Activated B-cell–like
AID: Activation-induced cytidine deaminase
BCR: B-cell receptor
CDR: Complementarity determining region
CLL: Chronic lymphoid leukemia
CSR: Class-switch recombination
D: Diversity
DLBCL: Diffuse large B-cell lymphoma
FC: Frequency classification
FW3: Framework region 3
GCB: Germinal-center B-cell–like
GEP: Gene expression profiling
HCDR3: Heavy chain CDR3
HLA: Histocompatibility antigen
IG: Immunoglobulin
IGH: Immunoglobulin heavy chain
IGK/L: Immunoglobulin kappa or lambda light chain
IGV: Immunoglobulin variable region gene
IMGT: International ImMunoGeneTics Information System
J: Joining
MHC: Major histocompatibility complex
mIHC: Multiplex immunohistochemistry
*MYC*/*BCL2*-R: *MYC*/*BCL2* translocation
NGS: Next-generation sequencing
NNE: Network ensembles
OS: Overall survival
PD-1: Programmed cell death protein 1
PD-L1: PD-1-ligand 1
PFS: Progression-free survival
pMHC: MHC-bound peptide
SHM: Somatic hypermutation
TCEM: T-cell exposed motif
TCR: T-cell receptor
Th2: Type 2 helper T cells
TLR9: Toll-like receptor 9
