# Supplementary material for: Immunoglobulin somatic hypermutation has clinical impact in DLBCL and potential implications for immune checkpoint blockade and neoantigen-based immunotherapies
Source: J Immunother Cancer. 2019 Oct 22;7:272. doi: 10.1186/s40425-019-0730-x (PMC6806565; doi:10.1186/s40425-019-0730-x)
Supplement: Supplementary file 1 — Additional file 1: Fig S1.. Construction and clinical outcome of the diffuse large B-cell lymphoma (DLBCL) cohort. Fig. S2. Diagram showing numbers of cases in this mutation study that have been characterized by various biomarker studies, and survival rates of patients whose sequencing results were correlated with prognosis. Fig. S3. CONSORT flow diagram illustrating the number of cases performed for high-throughput IG sequencing and clonal sequence analysis. Fig. S4. Molecular characterization for immunoglobulin heavy chain (IGH) gene usage in the study cohort. Fig. S5. Immunoglobulin heavy chain V gene (IGHV) somatic hypermutation (SHM) analysis. Fig. S6. Analysis for length of heavy chain CDR3. Fig. S7. Prediction of MHC-binding peptides and frequency of T-cell exposed motifs (TCEM) for immunoglobulin diagnostic sequences in the training set and validation set. (a) Regional distribution of relatively rare neoantigens derived from heavy chain and light chain immunoglobulin genes in DLBCL patients in the training set (top) and validation set (bottom). (b) Cases with high degree of heavy chain or light chain IGV SHM compared with cases without had higher frequency of relatively rare TCEM in the training (left) and validation sets (right). (c) In ABC-DLBCL, high IGV SHM was associated with lower tissue cellularity of CD4+ T cells. Fig. S8. Moleclar analysis for immunoglobulin heavy chain ongoing SHM and light chain SHM. Fig. S9. Immunoglobulin light chain SHM and CDR3 analysis. Fig S10. Comparison between different subsets of DLBCL. Fig S11. Light chain IGK/LV ongoing SHM analysis. [file 40425_2019_730_MOESM1_ESM.pdf]

Additional file 1 for

Immunoglobulin somatic hypermutation has clinical impact in DLBCL and potential implications for immune checkpoint blockade and neoantigen-based immunotherapies

Supplementary figure Legends

Figure S1. Construction and clinical outcome of the diffuse large B-cell lymphoma (DLBCL) cohort. (**a**) CONSORT flow diagram illustrating the construction of the training set and validation set comprising the overall study cohort. (**b**) Overall survival (OS) and progression-free survival (PFS) rates of the overall patients included in this study, and OS/ PFS comparison between germinal center B-cell-like (GCB) and activated B-cell-like (ABC) subtypes of DLBCL.

Figure S2. Diagram showing the numbers of cases in this mutation study that have been characterized by various biomarker studies, and survival rates of patients whose sequencing results were correlated with prognosis.

Figure S3. CONSORT flow diagram illustrating the number of cases performed for high-throughput IG sequencing and clonal sequence analysis. In the Venn diagram, green number is for cases with only heavy chain analyzed, red number is for cases with only light chain analyzed, and black number is for cases with both heavy and light chain analyzed. In the flowchart, green boxes are for heavy chain analysis, and red boxes are for light chain analysis.

Figure S4. Molecular characterization for immunoglobulin heavy chain (IGH) gene usage in the study cohort. (**a**) Stacked histograms for IGHD gene usage in cases with D-J-only (left) and cases with V-D-J (right) diagnostic sequences. (**b-c**) Stacked histograms showing the specific IGHV gene usage pattern of diagnostic clones in overall DLBCL and

germinal-center B-cell–like (GCB) and activated B-cell–like (ABC) DLBCL subtypes. IGHV4-34 is over-represented in the ABC-DLBCL cases compared with the GCB-DLBCL cases. Histogram showing the distribution of somatic mutations by V-gene family as measured by total reads in GCB-DLBCL and ABC-DLBCL.

Figure S5. Immunoglobulin heavy chain V gene (IGHV) somatic hypermutation (SHM) analysis. (**a**) Histogram showing the distribution of IGHV somatic mutations within productive diagnostic clonal sequences. The number above the bar chart is the absolute count of the total clones. GCB-DLBCL compared with ABC-DLBCL had a significantly higher mean level of IGHV SHM. (**b**) High degree of IGHV SHM (SHM^{high}) was associated with a nonsignificant trend of better progression-free survival (PFS) in overall DLBCL. (**c**) IGHV SHM^{high} was associated with significantly better overall survival (OS) in ABC-DLBCL. (**d**) In GCB-DLBCL, IGHV SHM^{high} was associated with better OS with borderline significance only in cases lacking *BCL2* rearrangement (*BCL2*-R) or *MYC* rearrangement (*MYC*-R) but not in cases with *BCL2*-R or *MYC*-R.

Figure S6. Analysis for length of heavy chain complementarity determining region 3 (HCDR3). (**a**) Long length of HCDR3 with a cutoff of 18 amino acids (aa) was associated with poorer progression-free survival (PFS) in DLBCL and ABC-DLBCL, and with poorer PFS and overall survival (OS) in GCB-DLBCL. (**b**) Short HCDR3 length was associated with significantly higher IGHV SHM degree in GCB-DLBCL, and higher IGK/L SHM degree in ABC-DLBCL. The cutoff of short length was < 16 aa in GCB-DLBCL and <18 aa in ABC-DLBCL. (**c**) Long HCDR3 length was associated with poorer OS in GCB-DLBCL cases of the validation set and ABC-DLBCL cases of the training set. (**d**) Long HCDR3 length was associated with significantly poorer PFS and OS in DLBCL in both the training and validation sets.

Figure S7. Prediction of MHC-binding peptides and frequency of T-cell exposed motifs (TCEM) for immunoglobulin diagnostic sequences in the training set and validation set. (**a**) Regional distribution of relatively rare neoantigens (TCEM FC>16) derived from heavy chain and light chain immunoglobulin genes in DLBCL patients in the training set (top) and validation set (bottom). Each dot represents one peptide predicted having high MHC-II-binding affinity (exceeding the -1 standard deviation threshold for MHC derived from 24 HLA-DR alleles) and relatively rare TCEM (FC>16). The color intensities of the dots are scaled on the FC scale from FC16 to the very rare FC24. (**b**) Cases with high degree of heavy chain or light chain IGV SHM compared with cases without had higher frequency of relatively rare TCEM (FC>16) in the training (left) and validation sets (right). (**c**) In ABC-DLBCL, high IGV SHM was associated with lower tissue cellularity of $CD4^+$ T cells.

Figure S8. Moleclar analysis for immunoglobulin heavy chain ongoing SHM and light chain SHM. (**a**) Histogram showing distribution of IGHV ongoing SHM over IGHV gene families. (**b**) High IGHV ongoing SHM was associated with *AICDA* upregulation in the validation set. High PD-L2 expression in macrophages (in overall cohort) and *PD-L1*/*PD-L2* gene amplification (in the ABC subtype) were associated with a higher mean percentage of subclones with IGHV ongoing SHM in the sequence repertoire. (**c**) Histogram showing distribution of IGK/LV SHM within light chain diagnostic clonal sequences. (**d**) Compared with IGKV SHM, IGLV SHM showed more correlation with IGHV SHM.

Figure S9. Immunoglobulin light chain SHM and CDR3 analysis. (**a**) IGK/LV SHM^{high} was associated with significantly worse progression-free survival (PFS) in GCB-DLBCL in both the training and validation sets. (**b-c**) High degree of IGK/LV SHM (SHM^{high}) was associated with significantly worse overall survival (OS) in GCB-DLBCL independent of *BCL2* and *MYC* rearrangement (R) status. The *P* value was most significant in cases with *BCL2* rearrangement. (**d**) A long immunoglobulin light chain kappa/lambda CDR3

(K/LCDR3) length was associated with poorer OS in overall DLBCL and ABC-DLBCL. (**e**) In GCB-DLBCL, IGK/LV SHM^{high} was significantly associated with higher *CTSL1* mRNA levels whereas IGHV SHM^{high} was significantly associated with lower *CTSF* mRNA levels. (**f**) In ABC-DLBCL, IGK/LV SHM^{high} was negatively associated with PD-1 expression in B cells, and positively associated with *AICDA* mRNA expression in the training set.

Figure S10. Comparison between different subsets of DLBCL. (**a**) The training set compared with the validation set had significantly higher mean levels of some *MHC* class II and cathepsin genes' mRNA expression. (**b**) In the validation set overall (and the GCB subtype, figures not shown), *MYC* rearrangement (*MYC*-R) was associated with downregulation of some *MHC* class II genes. In both the training and validation sets, *MYC*-R was significantly associated with *HLA-F* and *CTSH* downregulation (only figures for the entire cohort were shown). (**c**) In both the training and validation sets, ABC-DLBCL compared with GCB-DLBCL had significantly higher mean levels of macrophage- and CD8^{+} T cell-infiltration and PD-L1 expression on B cells whereas lower mean *HLA-DQB2* mRNA level (only figures for overall cohort were shown). In the overall cohort (and in the training set but not the validation set), ABC- compared with GCB-DLBCL had significantly higher mean level of *CTSL1* mRNA.

Figure S11. Light chain IGK/LV ongoing SHM analysis. (**a**) There were negative associations between IGV SHM and light chain ongoing SHM. Like light chain SHM^{high}, (shown in Figure 5d) IGHV SHM^{high} was associated with lower numbers of subclonal sequences with IGK/LV ongoing SHM. Conversely, high numbers of IGK/LV subclonal sequences with ongoing SHM were associated with lower mean levels of SHM in IGV. (**b**) Heatmap for gene signatures associated with high ongoing IGK/LV SHM in DLBCL and GCB-DLBCL. (**c**) In GCB-DLBCL, high IGK/LV ongoing SHM (≥17 subclones) was associated with significantly poorer overall survival (OS). The adverse prognostic effect of light chain ongoing SHM was not

significant in the validation set. (**d**) In the training set, high light chain IGV ongoing SHM was associated with significantly poorer survival.

Figure S1

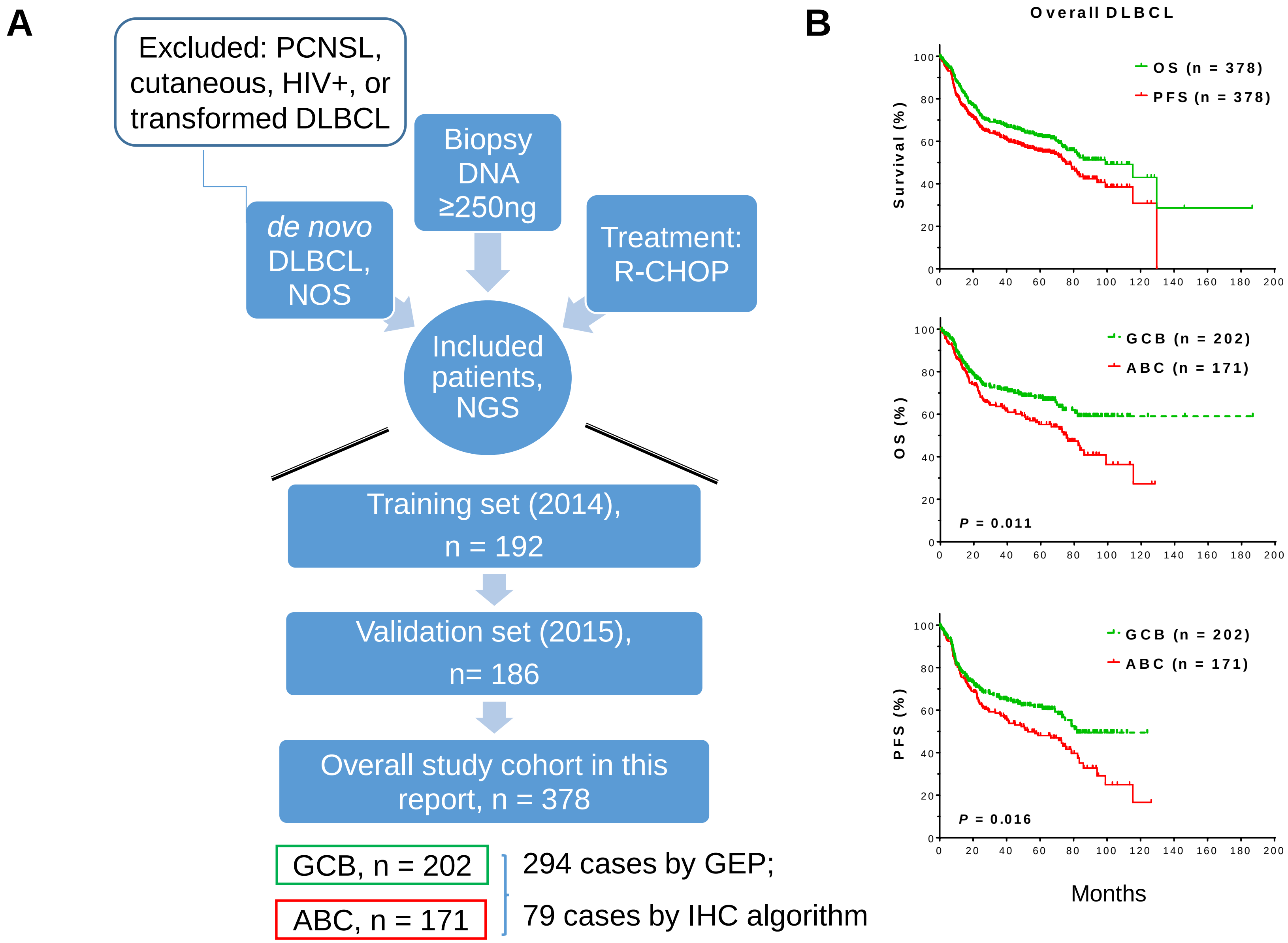

A
Excluded: PCNSL, cutaneous, HIV+, or transformed DLBCL
de novo DLBCL, NOS
Biopsy DNA ≥250ng
Treatment: R-CHOP
Included patients, NGS
Training set (2014), n = 192
Validation set (2015), n= 186
Overall study cohort in this report, n = 378
GCB, n = 202
ABC, n = 171
294 cases by GEP;
79 cases by IHC algorithm
B
Overall DLBCL
OS (n = 378)
PFS (n = 378)
Survival (%)
GCB (n = 202)
ABC (n = 171)
OS (%)
P = 0.011
GCB (n = 202)
ABC (n = 171)
PFS (%)
P = 0.016
Months

Figure S2

***IG* NGS n = 378**

n = 88: insufficient DNA sequencing reads or no expanded (>5%) clones → **Discard**

n = 290: index trackable sequences identified

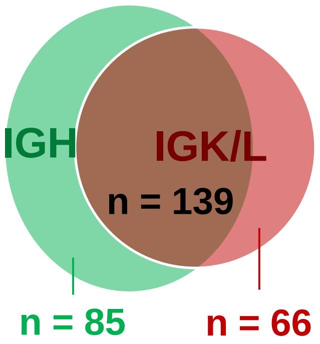

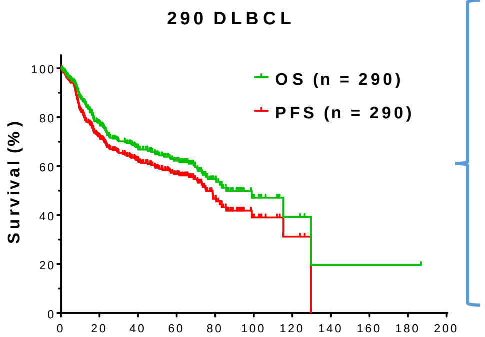

GCB, n = 146

ABC, n = 141

246 cases by gene expression profiling (112 ABC, 117 GCB, 17 UC);

58 cases by IHC algorithm (29 GC, 29 non-GC)

Other experiments

Mutiplex IHC for cell type-specific PD-1/L1/2 expression, n = 230;
FISH for *PDL1*, n = 235;
FISH for *PDL2*, n = 234;
FISH for *MYC*, n = 236;
FISH for *BCL2*, n = 256;
FISH for *BCL6*, n = 229;
FISH for *REL*, n = 221;
MYC mutation status, n = 268;
BCL6 5'UTR mutation status, n = 151;
TP53 mutation status, n = 263;
IHC for IgM, IgA, and IgG expression, n = 280;
IHC for Myc expression, n = 284;
IHC for Bcl-2 expression, n = 281;
IHC for CD37 expression, n = 273;
IHC for CXCR4 expression, n = 258;
IHC for CD30 expression, n = 284;
IHC for CD5 expression, n = 283;
IHC for p53 expression, n = 255;
IHC for p63 expression, n = 274;
IHC for MDM2 expression, n = 274;
IHC for Ki-67 expression, n = 276;
IHC for FOXP1 expression, n = 283;
IHC for PI3K expression, n = 263;
IHC for NF-κB p65 expression, n = 259;
IHC for NF-κB p50 expression, n = 255;
IHC for NF-κB p52 expression, n = 259

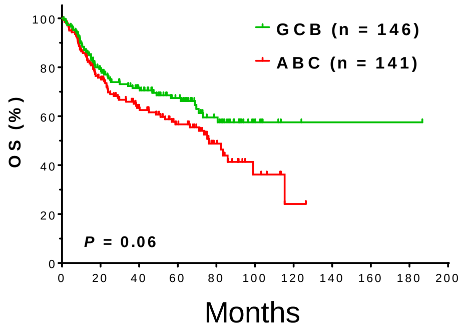

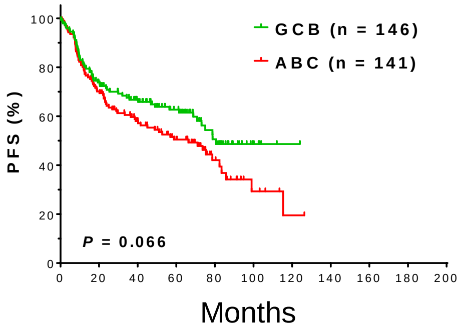

Figure S3

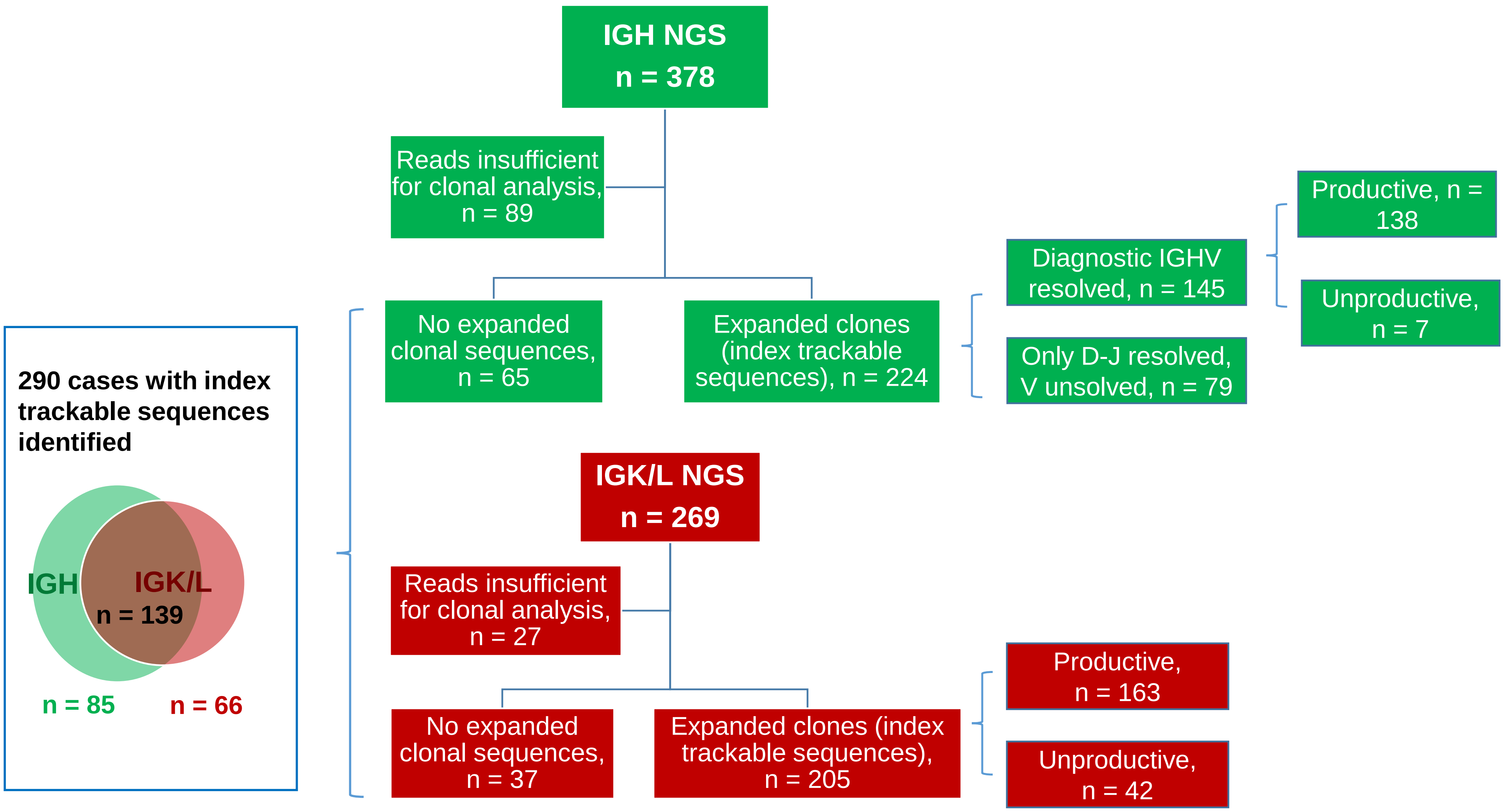
IGH NGS
n = 378
Reads insufficient for clonal analysis, n = 89
No expanded clonal sequences, n = 65
Expanded clones (index trackable sequences), n = 224
Diagnostic IGHV resolved, n = 145
Only D-J resolved, V unsolved, n = 79
Productive, n = 138
Unproductive, n = 7
IGK/L NGS
n = 269
Reads insufficient for clonal analysis, n = 27
No expanded clonal sequences, n = 37
Expanded clones (index trackable sequences), n = 205
Productive, n = 163
Unproductive, n = 42
290 cases with index trackable sequences identified
IGH
IGK/L
n = 139
n = 85
n = 66

Figure S4

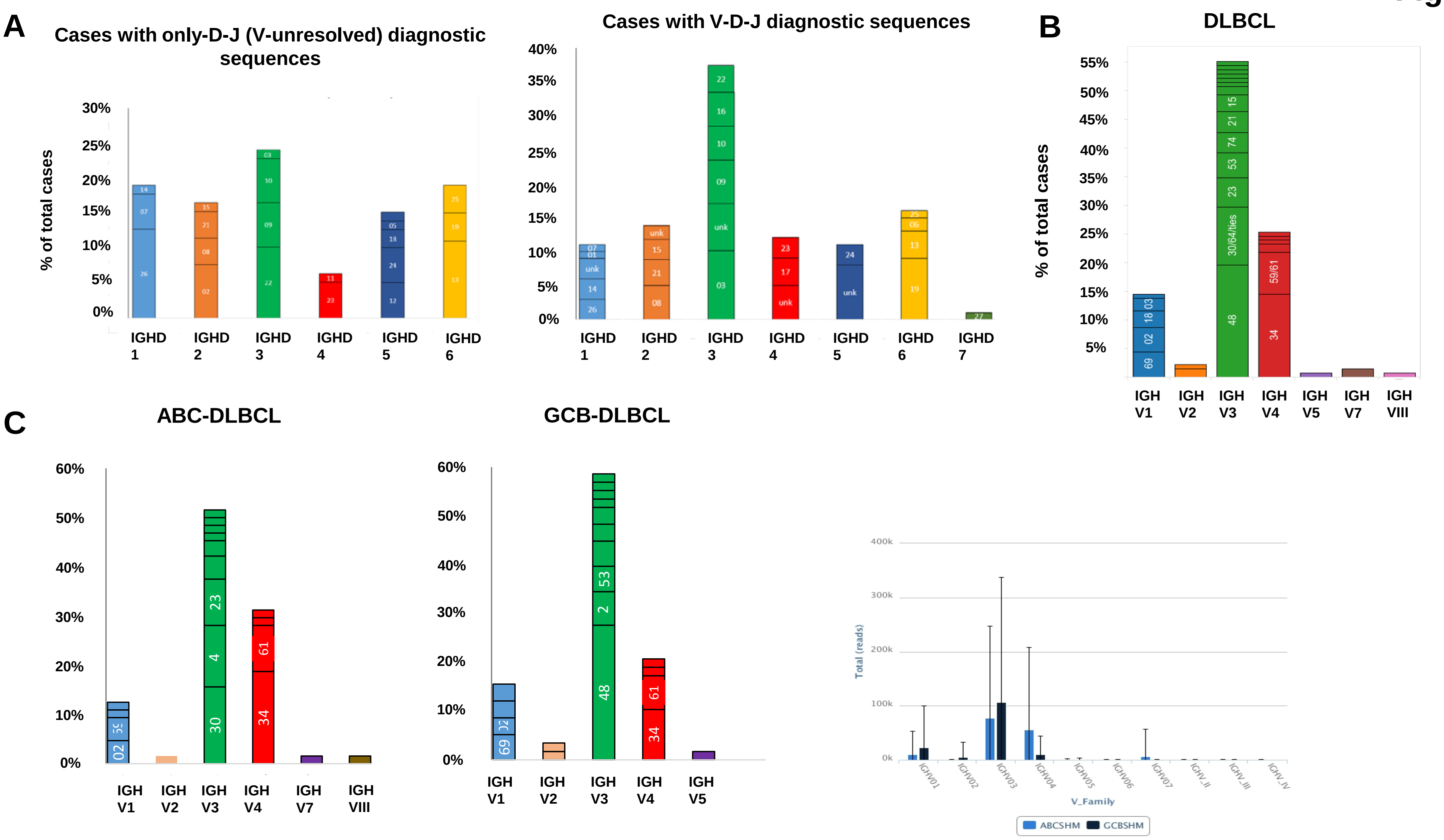
A
Cases with only-D-J (V-unresolved) diagnostic sequences
% of total cases
0%
5%
10%
15%
20%
25%
30%
IGHD 1
IGHD 2
IGHD 3
IGHD 4
IGHD 5
IGHD 6
Cases with V-D-J diagnostic sequences
0%
5%
10%
15%
20%
25%
30%
35%
40%
IGHD 1
IGHD 2
IGHD 3
IGHD 4
IGHD 5
IGHD 6
IGHD 7
B
DLBCL
% of total cases
5%
10%
15%
20%
25%
30%
35%
40%
45%
50%
55%
69
02
18
03
48
30/64/ties
23
53
74
21
15
34
59/61
IGH V1
IGH V2
IGH V3
IGH V4
IGH V5
IGH V7
IGH VIII
C
ABC-DLBCL
0%
10%
20%
30%
40%
50%
60%
02
30
4
23
34
61
IGH V1
IGH V2
IGH V3
IGH V4
IGH V7
IGH VIII
GCB-DLBCL
0%
10%
20%
30%
40%
50%
60%
69
48
2
53
34
61
IGH V1
IGH V2
IGH V3
IGH V4
IGH V5
Total (reads)
0k
100k
200k
300k
400k
IGHV01
IGHV02
IGHV03
IGHV04
IGHV05
IGHV06
IGHV07
IGHV_II
IGHV_III
IGHV_IV
V_Family
ABCSHM
GCBSHM

Figure S5

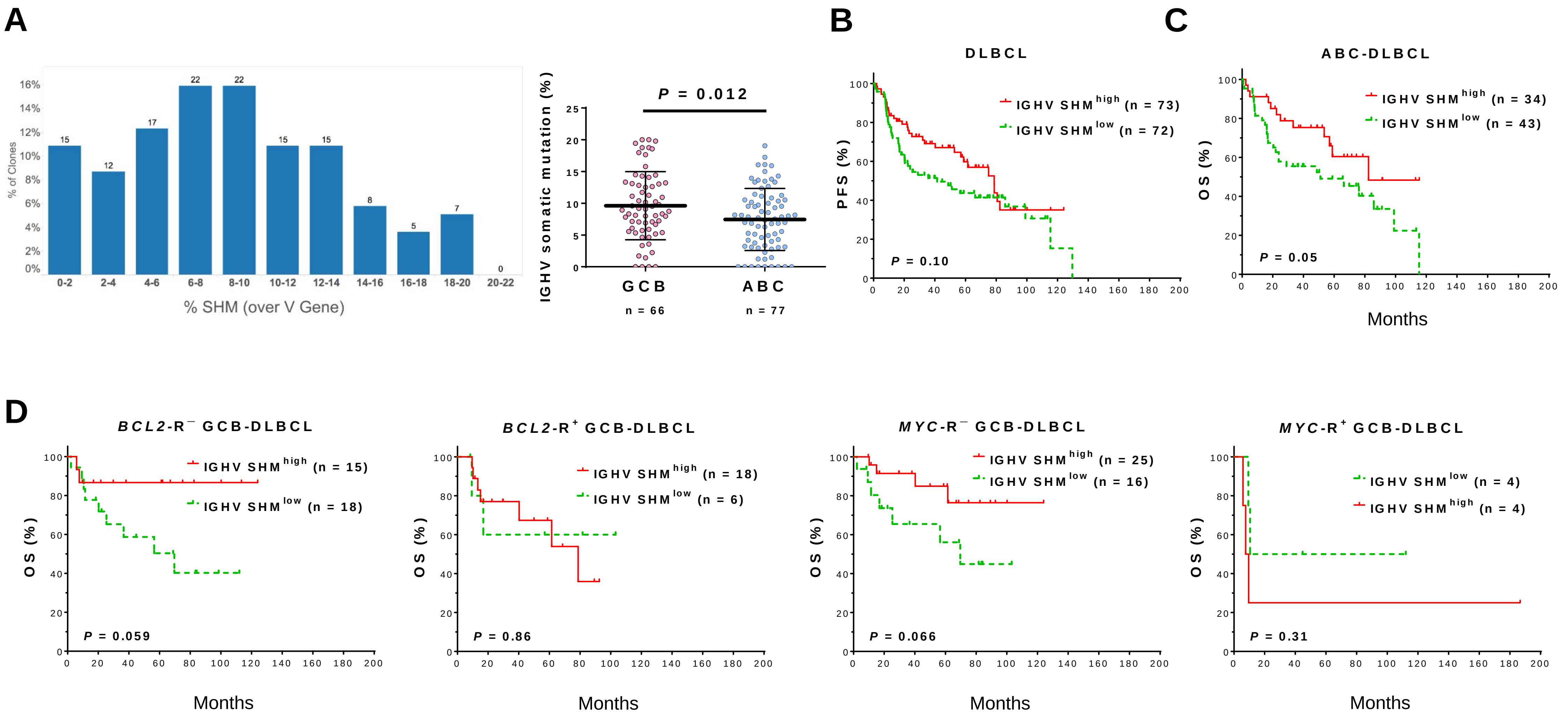
A
16%
14%
12%
10%
8%
6%
4%
2%
0%
% of Clones
15
12
17
22
22
15
15
8
5
7
0
0-2
2-4
4-6
6-8
8-10
10-12
12-14
14-16
16-18
18-20
20-22
% SHM (over V Gene)
IGHV somatic mutation (%)
P = 0.012
GCB
n = 66
ABC
n = 77
B
DLBCL
PFS (%)
IGHV SHM high (n = 73)
IGHV SHM low (n = 72)
P = 0.10
C
ABC-DLBCL
OS (%)
IGHV SHM high (n = 34)
IGHV SHM low (n = 43)
P = 0.05
Months
D
BCL2-R⁻ GCB-DLBCL
IGHV SHM high (n = 15)
IGHV SHM low (n = 18)
P = 0.059
BCL2-R⁺ GCB-DLBCL
IGHV SHM high (n = 18)
IGHV SHM low (n = 6)
P = 0.86
MYC-R⁻ GCB-DLBCL
IGHV SHM high (n = 25)
IGHV SHM low (n = 16)
P = 0.066
MYC-R⁺ GCB-DLBCL
IGHV SHM low (n = 4)
IGHV SHM high (n = 4)
P = 0.31
OS (%)
Months

Figure S6

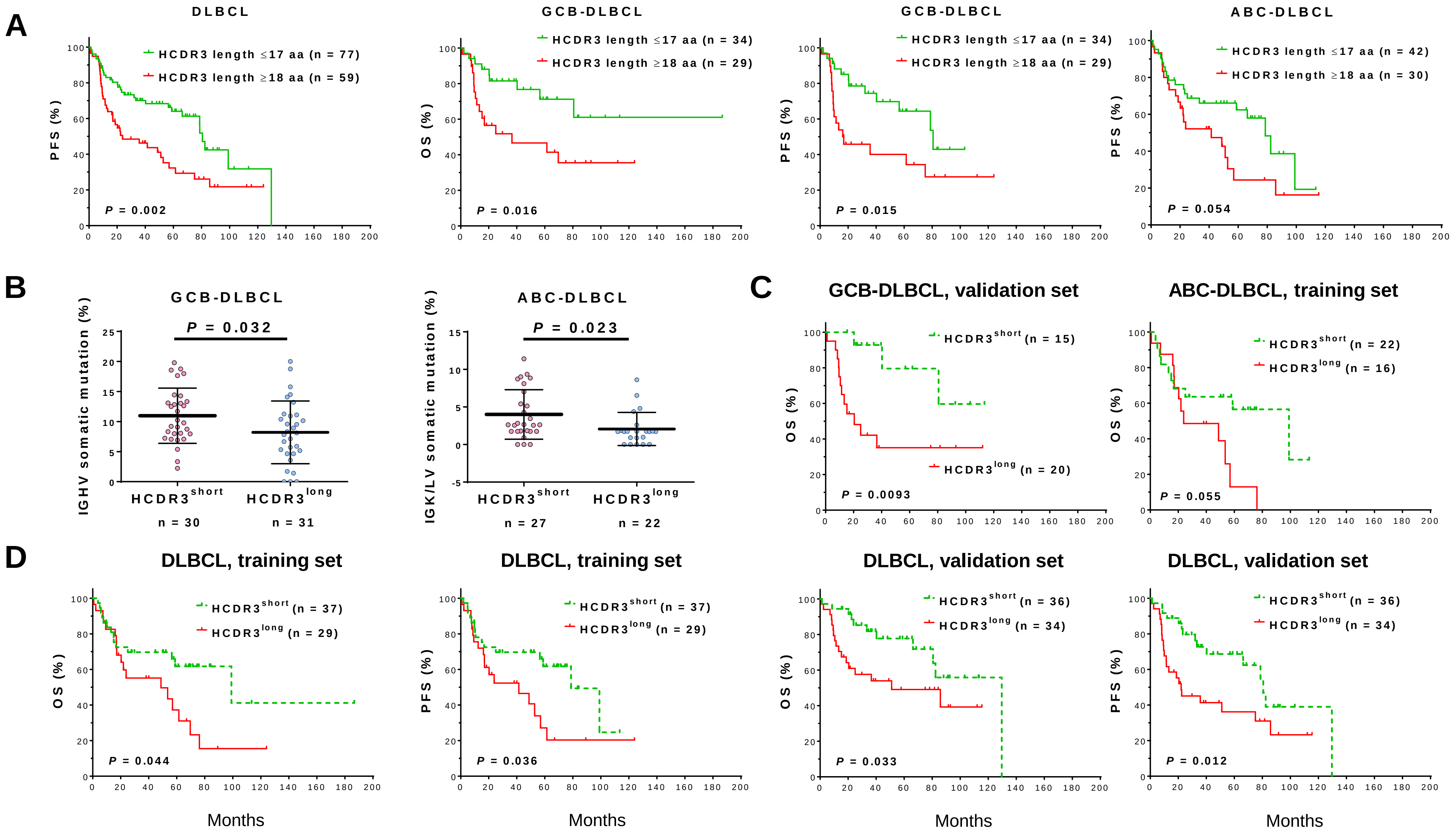

A
DLBCL
HCDR3 length ≤17 aa (n = 77)
HCDR3 length ≥18 aa (n = 59)
PFS (%)
P = 0.002
GCB-DLBCL
HCDR3 length ≤17 aa (n = 34)
HCDR3 length ≥18 aa (n = 29)
OS (%)
P = 0.016
GCB-DLBCL
HCDR3 length ≤17 aa (n = 34)
HCDR3 length ≥18 aa (n = 29)
PFS (%)
P = 0.015
ABC-DLBCL
HCDR3 length ≤17 aa (n = 42)
HCDR3 length ≥18 aa (n = 30)
PFS (%)
P = 0.054
B
GCB-DLBCL
P = 0.032
IGHV somatic mutation (%)
HCDR3^{short}
HCDR3^{long}
n = 30
n = 31
ABC-DLBCL
P = 0.023
IGK/LV somatic mutation (%)
HCDR3^{short}
HCDR3^{long}
n = 27
n = 22
C
GCB-DLBCL, validation set
HCDR3^{short} (n = 15)
HCDR3^{long} (n = 20)
OS (%)
P = 0.0093
ABC-DLBCL, training set
HCDR3^{short} (n = 22)
HCDR3^{long} (n = 16)
OS (%)
P = 0.055
D
DLBCL, training set
HCDR3^{short} (n = 37)
HCDR3^{long} (n = 29)
OS (%)
P = 0.044
DLBCL, training set
HCDR3^{short} (n = 37)
HCDR3^{long} (n = 29)
PFS (%)
P = 0.036
DLBCL, validation set
HCDR3^{short} (n = 36)
HCDR3^{long} (n = 34)
OS (%)
P = 0.033
DLBCL, validation set
HCDR3^{short} (n = 36)
HCDR3^{long} (n = 34)
PFS (%)
P = 0.012
Months
Months
Months
Months

Figure S7

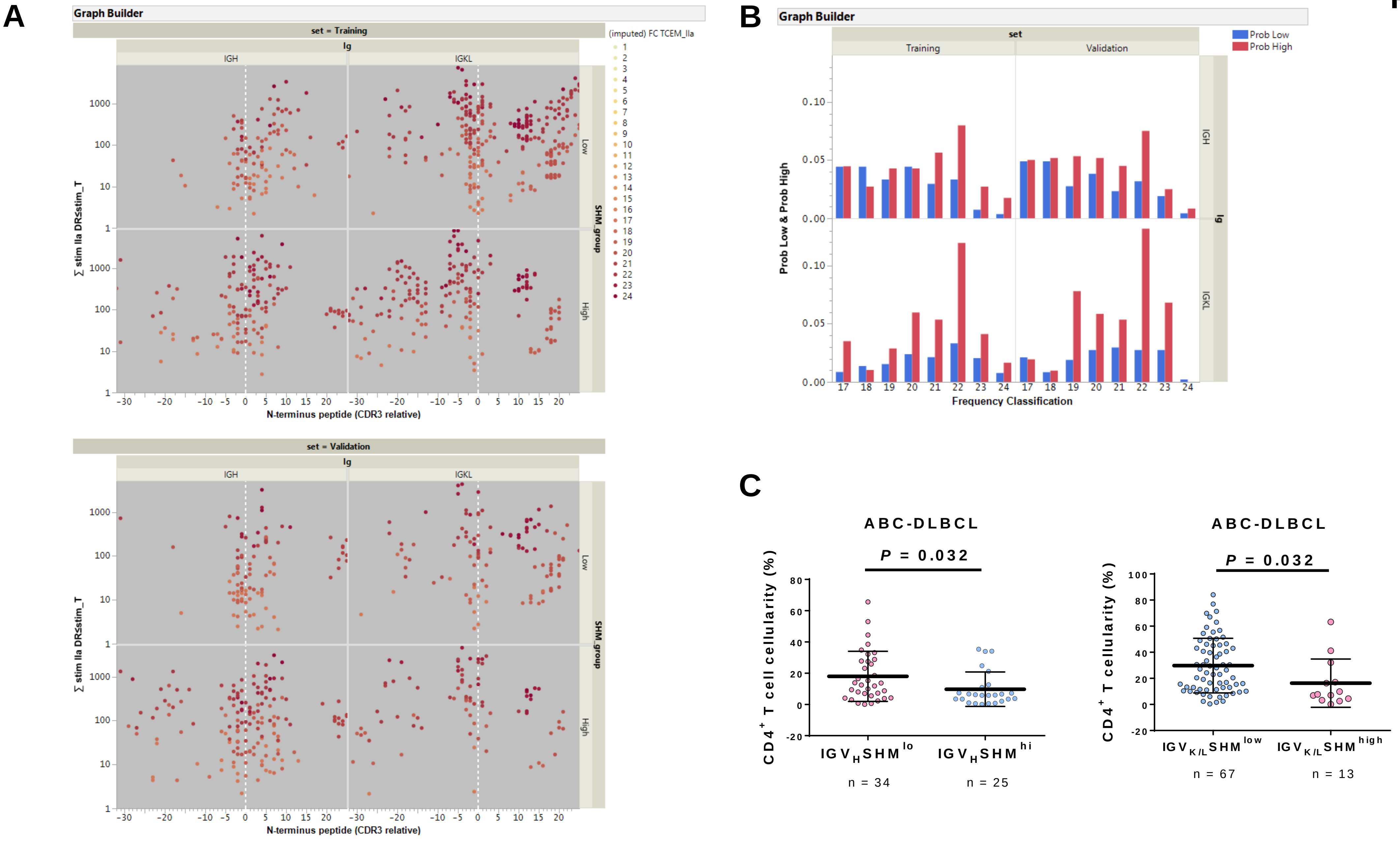
A
Graph Builder
set = Training
Ig
IGH
IGKL
(imputed) FC TCEM_IIa
Low
High
SHM_group
∑ stim IIa DR≤stim_T
N-terminus peptide (CDR3 relative)
set = Validation
B
Graph Builder
set
Training
Validation
Prob Low
Prob High
Prob Low & Prob High
Frequency Classification
IGH
IGKL
Ig
C
ABC-DLBCL
P = 0.032
CD4+ T cell cellularity (%)
IGVHSHMlo
IGVHSHMhi
n = 34
n = 25
IGVK/LSHMlow
IGVK/LSHMhigh
n = 67
n = 13

Figure S8

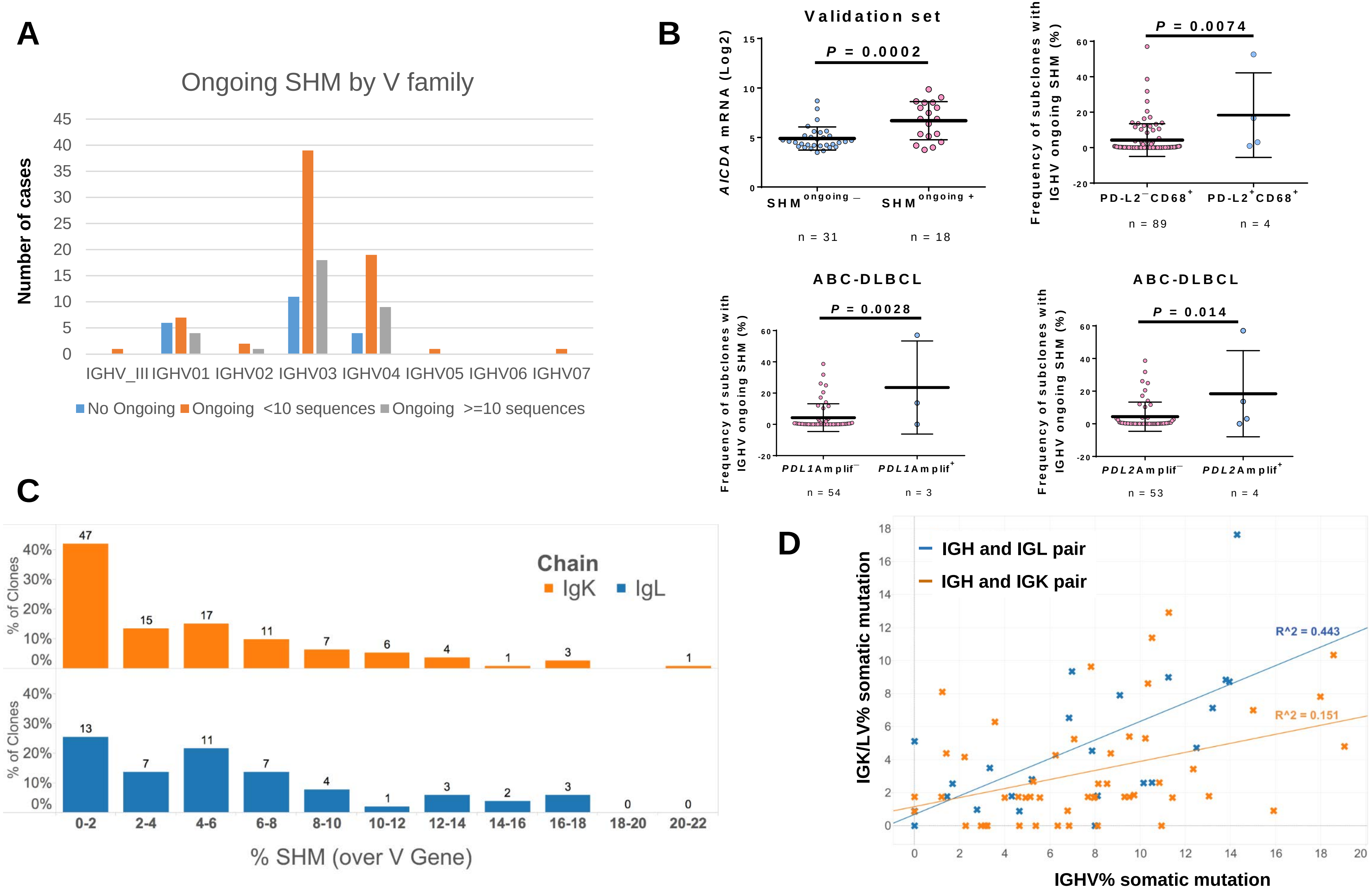
A
Ongoing SHM by V family
Number of cases
45
40
35
30
25
20
15
10
5
0
IGHV_III IGHV01 IGHV02 IGHV03 IGHV04 IGHV05 IGHV06 IGHV07
No Ongoing
Ongoing <10 sequences
Ongoing >=10 sequences
B
Validation set
P = 0.0002
AICDA mRNA (Log2)
SHM ongoing −
SHM ongoing +
n = 31
n = 18
P = 0.0074
Frequency of subclones with IGHV ongoing SHM (%)
PD-L2−CD68+
PD-L2+CD68+
n = 89
n = 4
ABC-DLBCL
P = 0.0028
PDL1Amplif−
PDL1Amplif+
n = 54
n = 3
ABC-DLBCL
P = 0.014
PDL2Amplif−
PDL2Amplif+
n = 53
n = 4
C
Chain
IgK
IgL
% of Clones
47
15
17
11
7
6
4
1
3
1
13
7
11
7
4
1
3
2
3
0
0
0-2
2-4
4-6
6-8
8-10
10-12
12-14
14-16
16-18
18-20
20-22
% SHM (over V Gene)
D
IGH and IGL pair
IGH and IGK pair
R^2 = 0.443
R^2 = 0.151
IGK/LV% somatic mutation
IGHV% somatic mutation

Figure S9

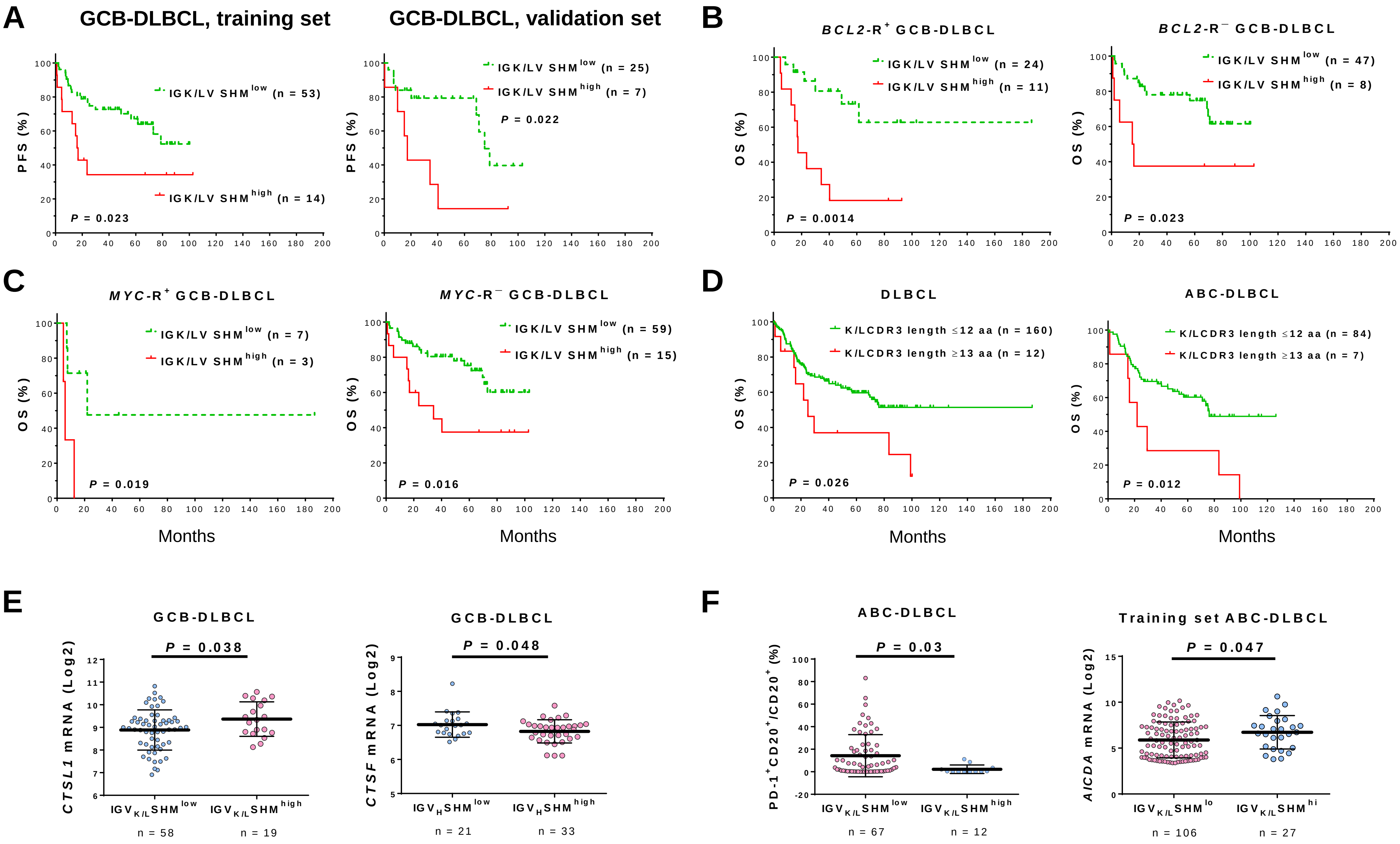
A
GCB-DLBCL, training set
PFS (%)
IGK/LV SHM low (n = 53)
IGK/LV SHM high (n = 14)
P = 0.023
GCB-DLBCL, validation set
IGK/LV SHM low (n = 25)
IGK/LV SHM high (n = 7)
P = 0.022
B
BCL2-R+ GCB-DLBCL
OS (%)
IGK/LV SHM low (n = 24)
IGK/LV SHM high (n = 11)
P = 0.0014
BCL2-R− GCB-DLBCL
IGK/LV SHM low (n = 47)
IGK/LV SHM high (n = 8)
P = 0.023
C
MYC-R+ GCB-DLBCL
IGK/LV SHM low (n = 7)
IGK/LV SHM high (n = 3)
P = 0.019
MYC-R− GCB-DLBCL
IGK/LV SHM low (n = 59)
IGK/LV SHM high (n = 15)
P = 0.016
D
DLBCL
K/LCDR3 length ≤12 aa (n = 160)
K/LCDR3 length ≥13 aa (n = 12)
P = 0.026
ABC-DLBCL
K/LCDR3 length ≤12 aa (n = 84)
K/LCDR3 length ≥13 aa (n = 7)
P = 0.012
Months
E
GCB-DLBCL
CTSL1 mRNA (Log2)
P = 0.038
IGVK/L SHM low
IGVK/L SHM high
n = 58
n = 19
GCB-DLBCL
CTSF mRNA (Log2)
P = 0.048
IGVH SHM low
IGVH SHM high
n = 21
n = 33
F
ABC-DLBCL
PD-1+CD20+/CD20+ (%)
P = 0.03
IGVK/L SHM low
IGVK/L SHM high
n = 67
n = 12
Training set ABC-DLBCL
AICDA mRNA (Log2)
P = 0.047
IGVK/L SHM lo
IGVK/L SHM hi
n = 106
n = 27

Figure S10

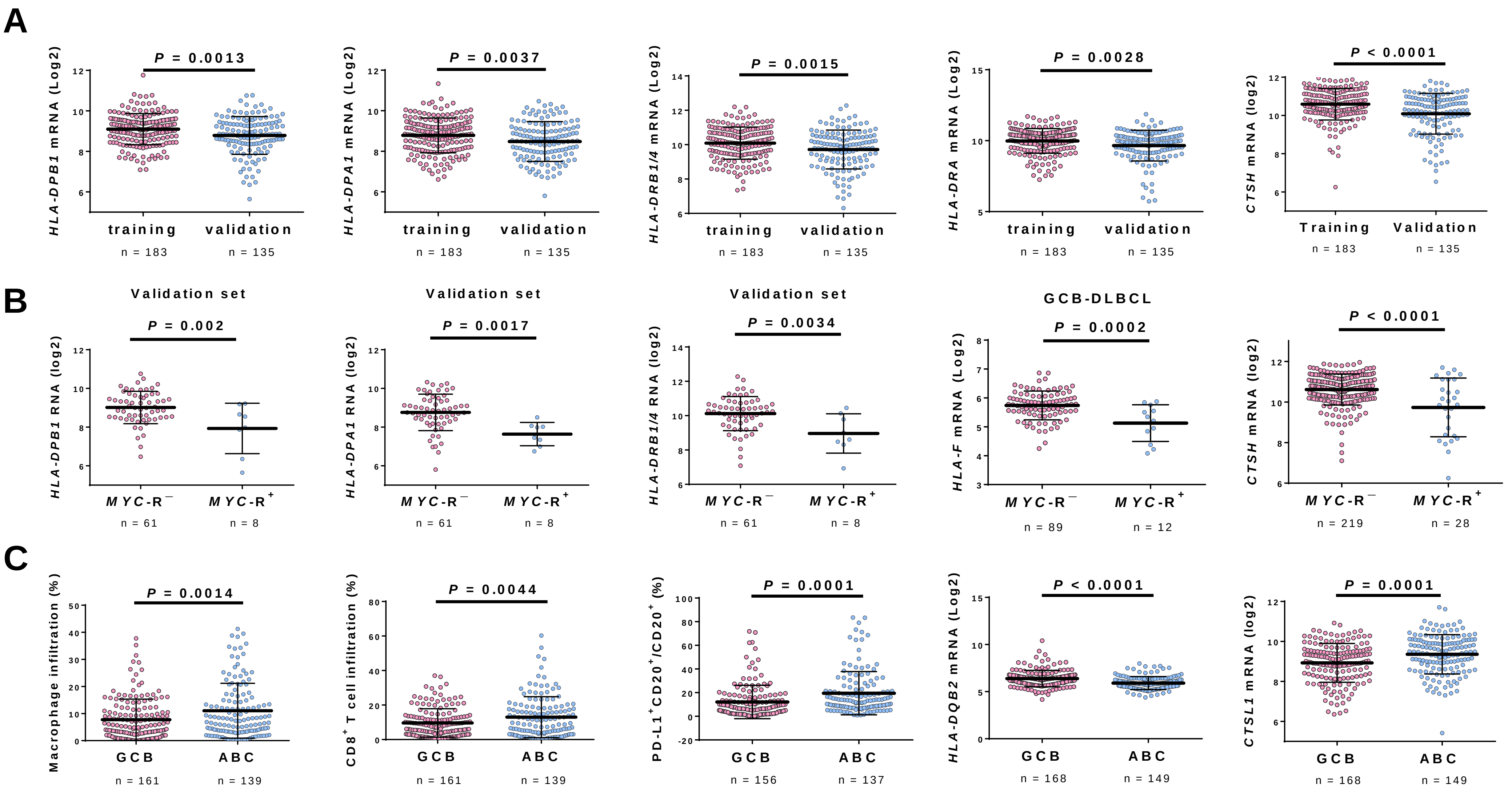

A
HLA-DPB1 mRNA (Log2)
P = 0.0013
training
validation
n = 183
n = 135
HLA-DPA1 mRNA (Log2)
P = 0.0037
HLA-DRB1/4 mRNA (Log2)
P = 0.0015
HLA-DRA mRNA (Log2)
P = 0.0028
CTSH mRNA (log2)
P < 0.0001
Training
Validation
B
Validation set
HLA-DPB1 RNA (log2)
P = 0.002
MYC-R⁻
MYC-R⁺
n = 61
n = 8
HLA-DPA1 RNA (log2)
P = 0.0017
HLA-DRB1/4 RNA (log2)
P = 0.0034
GCB-DLBCL
HLA-F mRNA (Log2)
P = 0.0002
n = 89
n = 12
CTSH mRNA (log2)
P < 0.0001
n = 219
n = 28
C
Macrophage infiltration (%)
P = 0.0014
GCB
ABC
n = 161
n = 139
CD8⁺ T cell infiltration (%)
P = 0.0044
PD-L1⁺CD20⁺/CD20⁺ (%)
P = 0.0001
n = 156
n = 137
HLA-DQB2 mRNA (Log2)
P < 0.0001
n = 168
n = 149
CTSL1 mRNA (log2)
P = 0.0001

Figure S11

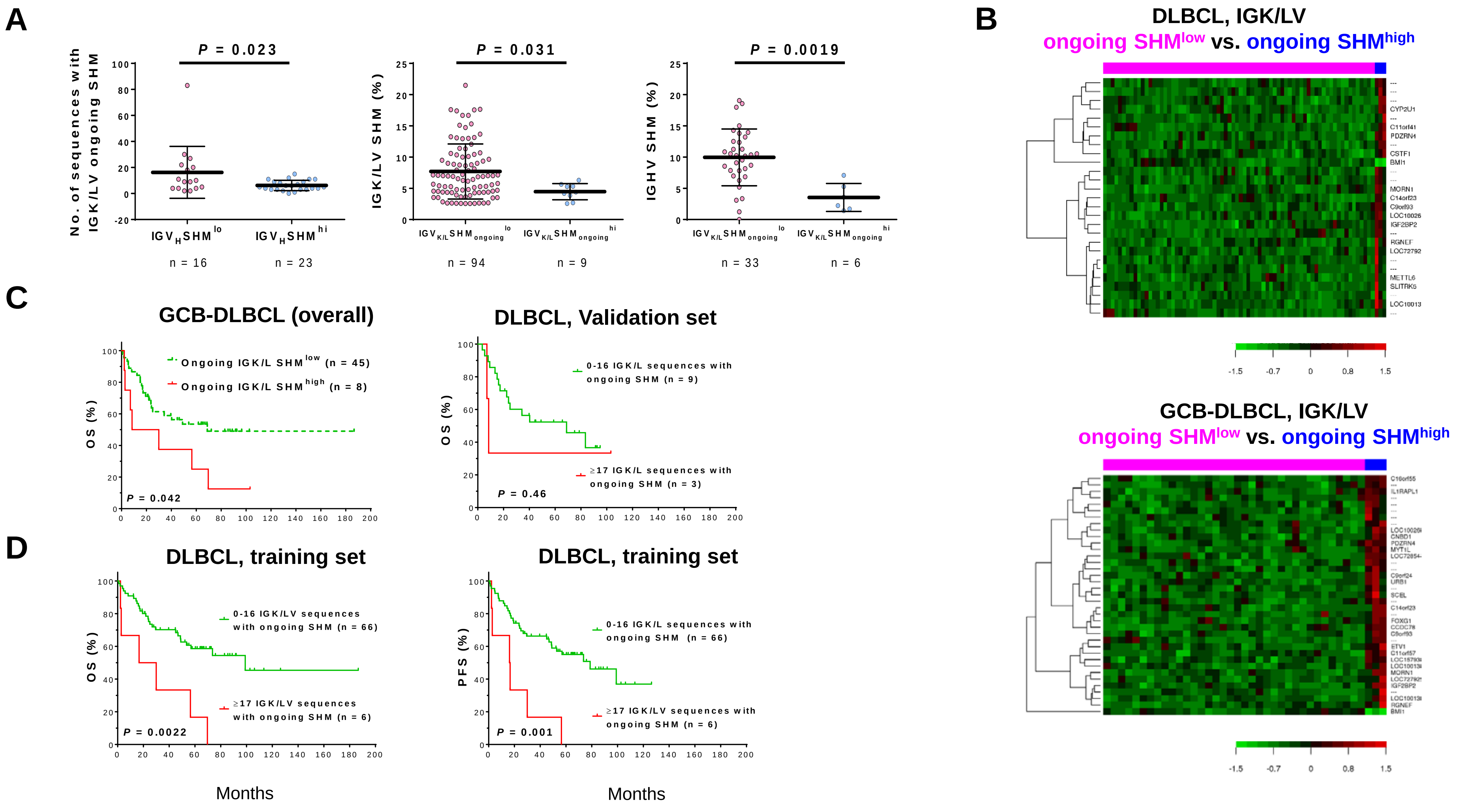

A
P = 0.023
No. of sequences with IGK/LV ongoing SHM
IGV H SHM lo
IGV H SHM hi
n = 16
n = 23
P = 0.031
IGK/LV SHM (%)
IGV K/L SHM ongoing lo
IGV K/L SHM ongoing hi
n = 94
n = 9
P = 0.0019
IGHV SHM (%)
n = 33
n = 6
B
DLBCL, IGK/LV
ongoing SHMlow vs. ongoing SHMhigh
GCB-DLBCL, IGK/LV
ongoing SHMlow vs. ongoing SHMhigh
-1.5
-0.7
0
0.8
1.5
C
GCB-DLBCL (overall)
Ongoing IGK/L SHM low (n = 45)
Ongoing IGK/L SHM high (n = 8)
OS (%)
P = 0.042
DLBCL, Validation set
0-16 IGK/L sequences with ongoing SHM (n = 9)
≥17 IGK/L sequences with ongoing SHM (n = 3)
P = 0.46
D
DLBCL, training set
0-16 IGK/LV sequences with ongoing SHM (n = 66)
≥17 IGK/LV sequences with ongoing SHM (n = 6)
P = 0.0022
DLBCL, training set
0-16 IGK/L sequences with ongoing SHM (n = 66)
≥17 IGK/LV sequences with ongoing SHM (n = 6)
PFS (%)
P = 0.001
Months
Months
