# Supplementary material for: Immunoglobulin somatic hypermutation has clinical impact in DLBCL and potential implications for immune checkpoint blockade and neoantigen-based immunotherapies
Source: J Immunother Cancer. 2019 Oct 22;7:272. doi: 10.1186/s40425-019-0730-x (PMC6806565; doi:10.1186/s40425-019-0730-x)
Supplement: Supplementary file 2 — Additional file 2: Table S1. Clinical features of 378 patients in the training and validation cohort whose DLBCL biopsies were sequenced and 290 patients whose sequencing results showed sufficient sequence reads. Table S2. Comparisons of clinicopathologic and molecular characteristics between patients with germinal-center B-cell–like (GCB) DLBCL with a low or high degree of somatic hypermutation (SHM) in immunoglobulin variable region genes. Table S3. Comparisons of clinicopathologic and molecular characteristics between patients with activated B-cell-like (ABC) subtype of DLBCL with a low or high degree of SHM in immunoglobulin variable region genes. Table S4. Significant prognostic effects of immunoglobulin molecular characteristics in DLBCL patients treated with R-CHOP by multivariate survival analysis. Table S5. Clinicopathologic and molecular characteristics of patients with DLBCL with a short or long immunoglobulin heavy/light chain CDR3 length. Table S6. Clinicopathologic and molecular characteristics of patients with DLBCL with ongoing SHM in immunoglobulin variable region genes. Table S7. Gene signatures associated with SHM in immunoglobulin sequences of DLBCL samples. Table S8. Multiple testing corrections for prognostic effects found in the overall cohort of DLBCL treated with R-CHOP by the Benjamini-Hochberg method with a false discovery rate of 0.10 [file 40425_2019_730_MOESM2_ESM.docx]

**Additional file 2 for**

### Immunoglobulin somatic hypermutation has clinical impact in DLBCL and potential implications for immune checkpoint blockade and neoantigen-based immunotherapies

**Table S1.** Clinical features of 378 patients in the training and validation cohort whose DLBCL biopsies were sequenced and 290 patients whose sequencing results showed sufficient sequence reads.

|  | **Overall study cohort** | **Training set** | **Validation set** | **Cases with results** |
| --- | --- | --- | --- | --- |
| **Characteristic** | **(n = 378)** | **(n = 192)** | **(n = 186)** | **(n = 290)** |
| **Age** |  |  |  |  |
| < 60 years | 158 | 75 | 82 | 118 |
| ≥ 60 years | 215 | 117 | 98 | 167 |
| Sex |  |  |  |  |
| Male | 217 | 111 | 106 | 163 |
| Female | 155 | 81 | 74 | 122 |
| **Stage** |  |  |  |  |
| I - II | 166 | 84 | 82 | 124 |
| III - IV | 192 | 100 | 92 | 151 |
| **B symptoms** |  |  |  |  |
| No | 230 | 129 | 101 | 177 |
| Yes | 126 | 52 | 74 | 93 |
| **Serum LDH levels** |  |  |  |  |
| Normal | 137 | 85 | 52 | 109 |
| Elevated | 193 | 83 | 110 | 146 |
| **No. of extranodal sites** | |  |  |  |
| 0 - 1 | 274 | 140 | 134 | 211 |
| ≥ 2 | 79 | 41 | 38 | 59 |
| **ECOG performance status** | |  |  |  |
| 0 - 1 | 264 | 143 | 121 | 201 |
| ≥ 2 | 60 | 22 | 38 | 43 |
| **Largest tumor size** |  |  |  |  |
| < 5cm | 176 | 88 | 88 | 129 |
| ≥ 5cm | 123 | 67 | 56 | 100 |
| **IPI score** |  |  |  |  |
| 0 - 2 | 219 | 115 | 104 | 167 |
| 3 - 5 | 137 | 69 | 68 | 105 |
| **Therapy response** |  |  |  |  |
| CR | 276 | 145 | 131 | 211 |
| PR | 53 | 28 | 25 | 40 |
| SD | 15 | 4 | 11 | 11 |
| PD | 28 | 15 | 13 | 22 |
| **GCB/ABC subtype** |  |  |  |  |
| GCB | 202 | 97 | 105 | 146 |
| ABC | 171 | 95 | 76 | 141 |

**Abbreviations:** IGH, immunoglobulin heavy chain gene; IGK/L, immunoglobulin kappa or lambda light chain gene; LDH, lactate dehydrogenase; ECOG, Eastern Cooperative Oncology Group; IPI, International Prognostic Index; CR, complete response; PR, partial response; SD, stable disease; PD, progressive disease.

**Table S2.** Comparisons of clinicopathologic and molecular characteristics between patients with germinal-center B-cell*–*like (GCB) diffuse large B-cell lymphoma (DLBCL) with a low or high degree of SHM in immunoglobulin variable region genes

|  |  |  | **GCB-DLBCL** |  |  |  |
| --- | --- | --- | --- | --- | --- | --- |
| **Characteristic** | **IGHV SHM^low^** | **IGHV SHM^high^** |  | **IGK/LV SHM^low^** | **IGK/LV SHM^high^** |  |
|  | n (%) | n (%) | *P* | n (%) | n (%) | *P* |
| **Age** |  |  |  |  |  |  |
| < 60 years | 12 (44%) | 17 (46%) | 1.0 | 36 (48%) | 9 (45%) | 1.0 |
| ≥ 60 years | 15 (56%) | 20 (54%) |  | 39 (52%) | 11 (55%) |  |
| **Sex** |  |  |  |  |  |  |
| Male | 16 (59%) | 19 (51%) | .62 | 43 (57%) | 12 (60%) | 1.0 |
| Female | 11 (41%) | 18 (49%) |  | 32 (43%) | 8 (40%) |  |
| **Stage** |  |  |  |  |  |  |
| I - II | 12 (48%) | 20 (56%) | .61 | 38 (53%) | 8 (40%) | .45 |
| III - IV | 13 (52%) | 16 (44%) |  | 34 (47%) | 12 (60%) |  |
| **B symptoms** |  |  |  |  |  |  |
| No | 15 (60%) | 24 (65%) | .79 | 52 (74%) | 15 (75%) | 1.0 |
| Yes | 10 (40%) | 13 (35%) |  | 18 (26%) | 5 (25%) |  |
| **Serum LDH level** | |  |  |  |  |  |
| Normal | 10 (43%) | 17 (47%) | .80 | 36 (55%) | 10 (56%) | 1.0 |
| Elevated | 13 (57%) | 19 (53%) |  | 30 (45%) | 8 (44%) |  |
| **No. of extranodal sites** | |  |  |  |  |  |
| 0 or 1 | 20 (83%) | 28 (80%) | 1.0 | 59 (83%) | 13 (68%) | .20 |
| ≥ 2 | 4 (17%) | 7 (20%) |  | 12 (17%) | 6 (32%) |  |
| **ECOG performance status** | | |  |  |  |  |
| 0 or 1 | 18 (90%) | 24 (77%) | .45 | 53 (83%) | 12 (71%) | .31 |
| ≥ 2 | 2 (10%) | 7 (23%) |  | 11 (17%) | 5 (29%) |  |
| **Largest tumor size** | |  |  |  |  |  |
| < 5 cm | 13 (65%) | 16 (55%) | .56 | 35 (57%) | 5 (33%) | .15 |
| ≥ 5 cm | 7 (35%) | 13 (45%) |  | 26 (43%) | 10 (67%) |  |
| **IPI score** | |  |  |  |  |  |
| 0 - 2 | 16 (64%) | 26 (72%) | .58 | 49 (68%) | 11 (55%) | .30 |
| 3 - 5 | 9 (36%) | 10 (28%) |  | 23 (32%) | 9 (45%) |  |
| **Therapy response** | |  |  |  |  |  |
| CR | 19 (70%) | 28 (76%) | .78* | 57 (76%) | 11 (55) | .093* |
| PR | 4 | 4 |  | 9 | 31 |  |
| SD | 1 | 2 |  | 3 | 1 |  |
| PD | 3 | 3 |  | 6 | 6 |  |
| ***BCL2* translocation** | |  |  |  |  |  |
| No | 18 (75%) | 15 (45%) | **.032** | 47 (66%) | 8 (42%) | .068 |
| Yes | 6 (25%) | 18 (55) |  | 24 (34%) | 11 (58%) |  |
| **MDM2 expression** | |  |  |  |  |  |
| < 40% | 19 (70%) | 34 (97%) | **.0075** | 53 (74%) | 13 (65%) | .57 |
| ≥ 40% | 8 (30%) | 1 (3%) |  | 19 (26%) | 7 (35%) |  |
| **p53 expression** | |  |  |  |  |  |
| < 20% | 12 (50%) | 25 (78%) | **.045** | 36 (53%) | 9 (50%) | 1.0 |
| ≥ 20% | 12 (50%) | 7 (22%) |  | 32 (47%) | 9 (50%) |  |
| **No. of IGK/LV sequences with ongoing SHM** | | | |  |  |  |
| 0 to 16 | 2 (29%) | 8 (100%) | **.007** | 24 (75%) | 21 (100%) | **.016** |
| ≥ 17 | 5 (71%) | 0 (0%) |  | 8 (25%) | 0 (0%) |  |

**Abbreviations:** IGHV, immunoglobulin heavy chain variable region gene; IGK/LV, immunoglobulin kappa or lambda light chain variable region gene; SHM^low^, low degree of SHM; SHM^high^, high degree of SHM; LDH, lactate dehydrogenase; ECOG, Eastern Cooperative Oncology Group; IPI, International Prognostic Index; CR, complete response; PR, partial response; SD, stable disease; PD, progressive disease.

**Note:** Not all patients had data available. Significant *P* values (Fisher’s exact test) are in bold. *For therapy response, *P* values were for comparisons between CR and non-CR cases.

**Table S3.** Comparisons of clinicopathologic and molecular characteristics between patients with activated B-cell-like (ABC) subtype of diffuse large B-cell lymphoma (DLBCL) with a low or high degree of somatic hypermutation (SHM) in immunoglobulin variable region genes

|  |  |  | **ABC-DLBCL** |  |  |  |
| --- | --- | --- | --- | --- | --- | --- |
| **Characteristic** | **IGHV SHM^low^** | **IGHV SHM^high^** |  | **IGK/LV SHM^low^** | **IGK/LV SHM^high^** |  |
|  | n (%) | n (%) | *P* | n (%) | n (%) | *P* |
| **Age** |  |  |  |  |  |  |
| < 60 years | 18 (42%) | 8 (24%) | .14 | 29 (33%) | 4 (25%) | .58 |
| ≥ 60 years | 25 (58%) | 25 (76%) |  | 58 (67%) | 12 (75%) |  |
| **Sex** |  |  |  |  |  |  |
| Male | 19 (44%) | 22 (67%) | .065 | 51 (59%) | 13 (81%) | .10 |
| Female | 24 (56%) | 11 (33%) |  | 36 (41%) | 3 (19%) |  |
| **Stage** |  |  |  |  |  |  |
| I - II | 16 (38%) | 14 (47%) | .48 | 34 (40%) | 5 (33%) | .78 |
| III - IV | 26 (62%) | 16 (53%) |  | 51 (60%) | 10 (67%) |  |
| **B symptoms** |  |  |  |  |  |  |
| No | 22 (52%) | 19 (61%) | .48 | 50 (59%) | 12 (80%) | .15 |
| Yes | 20 (48%) | 12 (39%) |  | 35 (41%) | 3 (20%) |  |
| **Serum LDH level** | |  |  |  |  |  |
| Normal | 11 (28%) | 12 (43%) | .30 | 31 (40%) | 7 (50%) | .56 |
| Elevated | 28 (72%) | 16 (57%) |  | 47 (60%) | 7 (50%) |  |
| **No. of extranodal sites** | |  |  |  |  |  |
| 0 or 1 | 30 (71%) | 25 (83%) | .27 | 66 (79%) | 11 (69%) | .52 |
| ≥ 2 | 12 (29%) | 5 (17%) |  | 18 (21%) | 5 (31%) |  |
| **ECOG performance status** | | |  |  |  |  |
| 0 or 1 | 28 (74%) | 20 (74%) | 1.0 | 59 (76%) | 12 (92%) | .28 |
| ≥ 2 | 10 (26%) | 7 (26%) |  | 19 (24%) | 1 (8%) |  |
| **Largest tumor size** | |  |  |  |  |  |
| < 5 cm | 23 (66%) | 11 (50%) | .28 | 38 (57%) | 6 (46%) | .55 |
| ≥ 5 cm | 12 (34%) | 11 (50%) |  | 29 (43%) | 7 (54%) |  |
| **IPI score** | |  |  |  |  |  |
| 0 - 2 | 23 (56%) | 16 (53%) | 1.0 | 47 (56%) | 8 (53%) | 1.0 |
| 3 - 5 | 18 (44%) | 14 (47%) |  | 37 (44%) | 7 (47%) |  |
| **Therapy response** | |  |  |  |  |  |
| CR | 30 (70%) | 26 (79%) | .44* | 68 (78%) | 15 (94%) | .19* |
| PR | 6 | 4 |  | 12 | 1 |  |
| SD | 1 | 1 |  | 1 | 0 |  |
| PD | 6 | 2 |  | 6 | 0 |  |
| ***MYC* translocation** | |  |  |  |  |  |
| No | 33 (87%) | 30 (100%) | .062 | 75 (90%) | 14 (93%) | 1.0 |
| Yes | 5 (13%) | 0 (0%) |  | 8 (10%) | 1 (7%) |  |
| **MYC mutation** | |  |  |  |  |  |
| No | 36 (90%) | 36 (95%) | .68 | 77 (92%) | 11 (73%) | **.04** |
| Yes | 4 (10%) | 2 (5%) |  | 7 (8%) | 4 (27%) |  |
| **PI3K expression** | |  |  |  |  |  |
| < 70% | 27 (69%) | 25 (78%) | .43 | 61 (73%) | 5 (31%) | **.0025** |
| ≥ 70% | 12 (31%) | 7 (22%) |  | 22 (27%) | 11 (69%) |  |
| **p63 expression** | |  |  |  |  |  |
| < 10% | 28 (65%) | 17 (52%) | .25 | 50 (58%) | 4 (25%) | **.027** |
| ≥ 10% | 15 (35%) | 16 (48%) |  | 36 (42%) | 12 (75%) |  |
| **PD-L1^+^CD20^+^ cells to PD-1^+^CD8^+^ T cells** | | |  |  |  |  |
| Far | 4 (13%) | 7 (32%) | .17 | 7 (11%) | 5 (42%) | **.018** |
| Close | 27 (87%) | 15 (68%) |  | 57 (89%) | 7 (58%) |  |

**Abbreviations:** IGHV, immunoglobulin heavy chain variable region gene; IGK/LV, **i**mmunoglobulin kappa or lambda light chain variable region gene; SHM^low^, low degree of SHM; SHM^high^, high degree of SHM; LDH, lactate dehydrogenase; ECOG, Eastern Cooperative Oncology Group; IPI, International Prognostic Index; CR, complete response; PR, partial response; SD, stable disease; PD, progressive disease.

**Note:** Not all patients had data available. Significant *P* values (Fisher’s exact test) are in bold. *For therapy response, *P* values were for comparisons between CR and non-CR cases.

**Table S4.** Significant prognostic effects of immunoglobulin molecular characteristics in diffuse large B-cell lymphoma (DLBCL) patients treated with R-CHOP (rituximab with cyclophosphamide, doxorubicin, vincristine, and prednisone) by multivariate survival analysis

|  | |  | | **OS** | |  |  | **PFS** |  |
| --- | --- | --- | --- | --- | --- | --- | --- | --- | --- |
| **Variable** | | **HR** | **95% CI** | | ***P*** | | **HR** | **95% CI** | ***P*** |
| **ABC-DLBCL** | | | | |  | |  |  |  |
| IGHV SHM^high^ | 0.45 | | 0.17-1.24 | | 0.12 | | 0.35 | 0.13-0.94 | **0.036** |
| IPI score >2 | 4.53 | | 1.61-12.7 | | **0.004** | | 2.83 | 1.07-7.46 | **0.036** |
| Female sex | 1.45 | | 0.55-3.79 | | 0.45 | | 1.41 | 0.55-3.59 | 0.47 |
| Tumor size ≥5 cm | 2.29 | | 0.91-5.75 | | 0.079 | | 1.74 | 0.72-4.18 | 0.22 |
| B symptoms | 1.00 | | 0.40-2.51 | | 0.99 | | 1.50 | 0.62-3.63 | 0.37 |
| *MYC* rearrangement | 0.97 | | 0.18-5.27 | | 0.97 | | 1.01 | 0.19-5.22 | 0.99 |
| **DLBCL** |  | |  | |  | |  |  |  |
| IGHV ongoing SHM^+^ | 2.56 | | 1.32-4.97 | | **0.006** | | 1.96 | 1.04-3.69 | **0.036** |
| IPI score >2 | 2.49 | | 1.26-4.92 | | **0.009** | | 2.16 | 1.12-4.16 | **0.021** |
| Female sex | 1.46 | | 0.67-3.20 | | 0.34 | | 1.10 | 0.52-2.32 | 0.81 |
| Tumor size ≥5 cm | 2.41 | | 1.11-5.22 | | **0.026** | | 1.96 | 0.94-4.11 | 0.073 |
| B symptoms | 0.95 | | 0.48-1.90 | | 0.89 | | 1.01 | 0.52-1.95 | 0.98 |
| ABC subtype | 1.13 | | 0.59-2.17 | | 0.72 | | 1.06 | 0.56-1.98 | 0.84 |
| **GCB-DLBCL** |  | |  | |  | |  |  |  |
| IGK/LV SHM^high^ | 3.55 | | 1.44-8.77 | | **0.006** | | 2.64 | 1.11-6.30 | **0.028** |
| IPI score >2 | 2.50 | | 1.06-5.87 | | **0.036** | | 2.63 | 1.17-5.91 | **0.019** |
| Female sex | 0.22 | | 0.074-0.65 | | **0.006** | | 0.37 | 0.14-0.95 | 0.038 |
| Tumor size ≥5 cm | 4.50 | | 1.72-11.7 | | **0.002** | | 3.62 | 1.49-8.79 | **0.004** |
| B symptoms | 2.28 | | 0.89-5.84 | | 0.085 | | 2.02 | 0.82-5.00 | 0.13 |
| **GCB-DLBCL** |  | |  | |  | |  |  |  |
| Long HCDR3 | 3.55 | | 1.44-8.77 | | **0.026** | | 2.92 | 1.03-8.26 | **0.043** |
| IPI score >2 | 4.18 | | 1.58-11.0 | | **0.004** | | 2.95 | 1.22-7.11 | **0.016** |
| Female sex | 1.80 | | 0.52-6.25 | | 0.36 | | 1.17 | 0.39-3.50 | 0.78 |
| Tumor size ≥5 cm | 1.40 | | 0.35-5.59 | | 0.63 | | 1.32 | 0.41-4.30 | 0.64 |
| B symptoms | 2.42 | | 0.92-6.33 | | 0.073 | | 1.85 | 0.76-4.53 | 0.18 |
| **DLBCL** |  | |  | |  | |  |  |  |
| Long K/LCDR3 | 3.96 | | 1.34-11.7 | | **0.013** | | 2.83 | 0.97-8.20 | 0.056 |
| IPI score >2 | 2.84 | | 1.60-5.06 | | **<0.001** | | 2.22 | 1.30-3.80 | **0.004** |
| Female sex | 0.66 | | 0.38-1.15 | | 0.14 | | 0.80 | 0.48-1.34 | 0.40 |
| Tumor size ≥5 cm | 3.06 | | 1.75-5.34 | | **<0.001** | | 2.45 | 1.47-4.11 | **0.001** |
| B symptoms | 2.11 | | 1.21-3.67 | | **0.008** | | 1.95 | 1.15-3.30 | **0.013** |

**Abbreviations**: OS, overall survival; PFS, progression-free survival; HR, hazard ratio; CI, confidence interval; ABC, activated B-cell–like; IGHV, immunoglobulin heavy chain variable region gene; SHM, somatic hypermutation; IPI, International Prognostic Index; GCB, germinal center B-cell–like; IGK/LV, immunoglobulin kappa or lambda light chain variable region gene; CDR3, complementarity determining region 3.

**Note:** Significant *P* values (Cox regression model) are in bold.

**Table S5.** Clinicopathologic and molecular characteristics of patients with diffuse large B-cell lymphoma (DLBCL) with a short or long immunoglobulin heavy/light chain complementarity determining region 3 (CDR3) length

|  | **GCB-DLBCL** | |  | **DLBCL** | |  |
| --- | --- | --- | --- | --- | --- | --- |
|  | **HCDR3^short^** | **HCDR3^long^** |  | **K/LCDR3^short^** | **K/LCDR3^long^** |  |
| **Characteristic** | n (%) | n (%) | *P* | n (%) | n (%) | *P* |
| **Age** |  |  |  |  |  |  |
| < 60 years | 12 (41%) | 14 (44%) | 1.0 | 61 (39%) | 6 (46%) | .77 |
| ≥ 60 years | 17 (59%) | 18 (56%) |  | 96 (61%) | 7 (54%) |  |
| **Sex** |  |  |  |  |  |  |
| Male | 16 (55%) | 17 (53%) | 1.0 | 94 (60%) | 7 (54%) | .77 |
| Female | 13 (45%) | 15 (47%) |  | 63 (40%) | 6 (46%) |  |
| **Stage** |  |  |  |  |  |  |
| I - II | 8 (28%) | 19 (66%) | **.0079** | 66 (43%) | 6 (50%) | .77 |
| III - IV | 21 (72%) | 10 (34%) |  | 86 (57%) | 6 (50%) |  |
| **B symptoms** |  |  |  |  |  |  |
| No | 17 (59%) | 20 (67%) | .60 | 99 (66%) | 8 (73%) | .75 |
| Yes | 12 (41%) | 10 (33%) |  | 52 (34%) | 3 (27%) |  |
| **Serum LDH level** | |  |  |  |  |  |
| Normal | 17 (61%) | 6 (21%) | **.006** | 64 (45%) | 5 (50%) | .76 |
| Elevated | 11 (39%) | 22 (79%) |  | 79 (55%) | 5 (50%) |  |
| **No. of extranodal sites** | | |  |  |  |  |
| 0 or 1 | 22 (81%) | 24 (83%) | 1.0 | 117 (77%) | 10 (83%) | 1.0 |
| ≥ 2 | 5 (19%) | 5 (17%) |  | 35 (23%) | 2 (17%) |  |
| **ECOG performance status** | | |  |  |  |  |
| 0 or 1 | 18 (75%) | 22 (92%) | .24 | 109 (79%) | 7 (70%) | .45 |
| ≥ 2 | 6 (25%) | 2 (8%) |  | 29 (21%) | 3 (30%) |  |
| **Largest tumor size** | |  |  |  |  |  |
| <5 cm | 17 (74%) | 9 (38%) | **.019** | 74 (57%) | 4 (57%) | 1.0 |
| ≥5 cm | 6 (26%) | 15 (63%) |  | 56 (43%) | 3 (43%) |  |
| **IPI score** | |  |  |  |  |  |
| 0 - 2 | 19 (68%) | 19 (63%) | .79 | 90 (59%) | 9 (82%) | .20 |
| 3 - 5 | 9 (32%) | 11 (37%) |  | 62 (41%) | 2 (18%) |  |
| **Therapy response** | |  |  |  |  |  |
| CR | 24 (83%) | 18 (56%) | **.03*** | 114 (73%) | 7 (54%) | .20* |
| PR | 1 | 7 |  | 21 | 4 |  |
| SD | 1 | 2 |  | 6 | 0 |  |
| PD | 3 | 5 |  | 16 | 2 |  |
| **CD37 expression** | |  |  |  |  |  |
| No | 15 (56%) | 26 (87%) | **.017** | 97 (63%) | 7 (58%) | .76 |
| Yes | 12 (44%) | 4 (13%) |  | 56 (37%) | 5 (42%) |  |
| ***MYC* gene mutations** | |  |  |  |  |  |
| No | 16 (55%) | 23 (82%) | **.045** | 110 (72%) | 9 (69%) | .76 |
| Yes | 13 (45%) | 5 (18%) |  | 42 (28%) | 4 (31%) |  |
| **Kappa/Lambda** |  |  |  |  |  |  |
| Lambda | 4 (22%) | 6 (38%) | .46 | 39 (27%) | 9 (69%) | **.0029** |
| Kappa | 14 (78%) | 10 (63%) |  | 107 (73%) | 4 (31%) |  |

**Abbreviations**: GCB, germinal center B-cell-like; LDH, lactate dehydrogenase; ECOG, Eastern Cooperative Oncology Group; IPI, International Prognostic Index; CR, complete response; PR, partial response; SD, stable disease; PD, progressive disease; HCDR3, immunoglobulin heavy chain complementarity determining region 3; K/LCDR3, immunoglobulin light chain (kappa or lambda type) complementarity determining region 3.

**Note:** Not all patients had data available. Significant *P* values (Fisher’s exact test) are in bold. *For therapy response, *P* values were for comparisons between CR and non-CR cases.

**Table S6.** Clinicopathologic and molecular characteristics of patients with diffuse large B-cell lymphoma (DLBCL) with ongoing somatic hypermutation (SHM) in immunoglobulin variable region genes

|  | **IGHV SHM_ongoing_^+^** | **IGHV SHM_ongoing_^−^** |  | **IGK/LV SHM_ongoing_^high^** | **IGK/LV SHM_ongoing_^low^** |  |
| --- | --- | --- | --- | --- | --- | --- |
| **Characteristic** | n (%) | n (%) | *P* | n (%) | n (%) | *P* |
| **Age** |  |  |  |  |  |  |
| < 60 years | 15 (39%) | 31 (38%) | 1.0 | 2 (22%) | 34 (37%) | .48 |
| ≥ 60 years | 23 (61%) | 51 (62%) |  | 7 (78%) | 58 (63%) |  |
| **Sex** |  |  |  |  |  |  |
| Male | 21 (55%) | 44 (54%) | 1.0 | 5 (56%) | 62 (67%) | .48 |
| Female | 17 (45%) | 38 (46%) |  | 4 (44%) | 30 (33%) |  |
| **Stage** |  |  |  |  |  |  |
| I - II | 13 (37%) | 41 (53%) | .16 | 3 (38%) | 38 (42%) | 1.0 |
| III - IV | 22 (63%) | 37 (47%) |  | 5 (63%) | 52 (58%) |  |
| **B symptoms** |  |  |  |  |  |  |
| No | 19 (54%) | 53 (66%) | .30 | 3 (38%) | 64 (73%) | .052 |
| Yes | 16 (46%) | 27 (34%) |  | 5 (63%) | 24 (27%) |  |
| **Serum LDH level** | |  |  |  |  |  |
| Normal | 18 (50%) | 26 (36%) | .21 | 0 (0%) | 47 (57%) | **.0021** |
| Elevated | 18 (50%) | 47 (64%) |  | 8 (100%) | 36 (43%) |  |
| **No. of extranodal sites** | | |  |  |  |  |
| 0 or 1 | 25 (74%) | 64 (82%) | .32 | 6 (67%) | 69 (78%) | .43 |
| ≥ 2 | 9 (26%) | 14 (18%) |  | 3 (33%) | 20 (22%) |  |
| **ECOG performance status** | | |  |  |  |  |
| 0 or 1 | 23 (70%) | 56 (82%) | .20 | 6 (75%) | 62 (77%) | 1.0 |
| ≥ 2 | 10 (30%) | 12 (18%) |  | 2 (25%) | 19 (23%) |  |
| **Largest tumor size** | |  |  |  |  |  |
| < 5 cm | 19 (63%) | 38 (61%) | 1.0 | 3 (43%) | 40 (53%) | .70 |
| ≥ 5 cm | 11 (37%) | 24 (39%) |  | 4 (57%) | 35 (47%) |  |
| **IPI score** | |  |  |  |  |  |
| 0 - 2 | 20 (56%) | 52 (68%) | .29 | 1 (13%) | 53 (60%) | **.020** |
| 3 - 5 | 16 (44%) | 25 (32%) |  | 7 (81%) | 35 (40%) |  |
| **Therapy response** | |  |  |  |  |  |
| CR | 28 (74%) | 58 (58%) | .83* | 3 (33%) | 71 (77%) | **.010*** |
| PR | 4 | 12 |  | 4 | 9 |  |
| SD | 0 | 5 |  | 0 | 4 |  |
| PD | 6 | 7 |  | 2 | 8 |  |
| **GCB/ABC subtype** | |  |  |  |  |  |
| GCB | 16 (43%) | 42 (49%) | .56 | 8 (89%) | 45 (48%) | **.032** |
| ABC | 21 (57%) | 43 (51%) |  | 1 (11%) | 48 (52%) |  |
| **BAGS classification** | |  |  |  |  |  |
| Centroblast | 12 (63%) | 10 (25%) | **.0086**^§^ | 4 (67%) | 19 (39%) | .22^§^ |
| Centrocyte | 7 (37%) | 30 (75%) |  | 2 (33%) | 30 (61%) |  |
| Others | 9 | 21 |  | 1 | 18 |  |
| **CD5 expression** | |  |  |  |  |  |
| No | 82 (96%) | 29 (78%) | **.003** | 8 (89%) | 86 (93%) | .49 |
| Yes | 3 (4%) | 8 (22%) |  | 1 (11%) | 6 (7%) |  |
| **IgM expression** | |  |  |  |  |  |
| < 20% | 19 (50%) | 59 (73%) | **.022** | 8 (89%) | 64 (73%) | .44 |
| ≥ 20% | 19 (50%) | 22 (27%) |  | 1 (11%) | 24 (27%) |  |
| **PD-L2^+^% of CD68^+^ cells** | |  |  |  |  |  |
| < 14.2% | 25 (83%) | 61 (97%) | **.034** | 8 (100%) | 64 (97%) | 1.0 |
| ≥ 14.2% | 5 (17%) | 2 (3%) |  | 0 (0%) | 2 (3%) |  |
| **9p24 locus by a *PDL1* probe** | |  |  |  |  |  |
| Normal/gain | 66 (97%) | 22 (85%) | **.048** | 72 (94%) | 4 (50%) | **.0036** |
| Amplification/polyploid | 2 (3%) | 4 (15%) |  | 5 (6%) | 4 (50%) |  |
| **9p24 locus by a *PDL2* probe** | |  |  |  |  |  |
| Normal/gain | 64 (97%) | 25 (83%) | **.029** | 71 (91%) | 4 (50%) | **.0082** |
| Amplification/polyploid | 2 (3%) | 5 (17%) |  | 7 (9%) | 4 (50%) |  |
| **SHM degree** |  |  |  |  |  |  |
| SHM^low^ | 32 (40%) | 19 (50%) | .19 | 56 (60%) | 9 (100%) | **.025** |
| SHM^high^ | 49 (60%) | 19 (50%) |  | 38 (40%) | 0 (0%) |  |

**Abbreviations**: IGHV, immunoglobulin heavy chain variable region gene; IGK/LV, **i**mmunoglobulin kappa or lambda light chain variable region gene; LDH, lactate dehydrogenase; ECOG, Eastern Cooperative Oncology Group; IPI, International Prognostic Index; CR, complete response; PR, partial response; SD, stable disease; PD, progressive disease; GCB, germinal center B-cell-like; ABC, activated B-cell–like; BAGS, B-cell–associated gene signature.

**Note:** Not all patients had data available. Significant *P* values (Fisher’s exact test) are in bold. *For therapy response, *P* values were for comparisons between CR and non-CR cases. ^§^Combined centroblast and centrocyte case numbers vs others.

**Table S7.** Gene signatures associated with SHM in immunoglobulin sequences of diffuse large B-cell lymphoma (DLBCL) samples

| **Comparison** | **Upregulated genes** | **Downregulated genes** |
| --- | --- | --- |
| High vs. low degree of IGHV SHM (FDR 0.35) | *BTG1, ATP2B1, C1orf71, ADRBK2, SYPL1, EEA1, USP34, APPL2, JMJD1C, ATXN3, BDH2, GARNL1, CHIC2, KIAA1715, RAB39B* | *ZNF667,* ***CD6****, CETP, ICAM5, SAP30, SYDE1, MFI2, ALDH1B1, C1QTNF6, GJB1,* ***WNT6****, LOC100134017, SLC1A7,* ***AKNA****, CHRNA4, SNX20, ST6GALNAC4, LOC390595****, IL27****, HR, LOC100291944, LOC100293662, DGCR14, ZNF564/ZNF709, PRRT1, B3GNT7, CNTN2, CABP5, KIAA1522, C1orf62* |
| Intraclonal sequences with ongoing IGHV SHM ≥2% the repertoire in ABC-DLBCL (FDR 0.3) | *SLC17A7, OR2H1, SLC14A2, NCRNA00105, GSG1, SPAG8,* ***EBF3****, HOXC10, DYRK1B, MAN1A2, LOC729652,* ***PCNA****, HYDIN/HYDIN2, ADCY8, C14orf56, IRX4, ZFYVE28, FAM38B, SCARNA17, INVS, C6orf105* | *COL4A1, SPARC, H3F3B, CMTM6, SLC35A4, VTA1, STK16, LMBR1* |
| High vs. low numbers of sequences with ongoing IGK/LV SHM (FDR 0.01) | *CYP2U1, C11orf41, PDZRN4, CSTF1, MORN1, C14orf23, C9orf93, LOC100268168, IGF2BP2, RGNEF, LOC727924, METTL6, SLITRK5, LOC100130958* | *BMI1* |
| High vs. low numbers of sequences with ongoing IGK/LV SHM in GCB-DLBCL (FDR 0.05) | *C14orf23, FOXG1, CCDC78, IGF2BP2, LOC728543, C11orf57, PDZRN4, C9orf24, MORN1, LOC100132077, LOC100130958, C16orf55, ETV1, RGNEF, C9orf93, LOC100268168, LOC727924, URB1, SCEL, LOC157931, MYT1L, CNBD1, IL1RAPL1* | *BMI1* |

**Abbreviations**: IGH, immunoglobulin heavy chain; ABC, activated B-cell–like; FDR, false discovery rate; IGHV*,* IGH variable region gene; SHM, somatic hypermutation; IGK/LV, immunoglobulin kappa or lambda light chain variable region gene; GCB, germinal center B-cell–like. Abbreviations for highlighted genes (in bold): *CD6*, CD6 molecule or T-cell differentiation antigen CD6; *WNT6,* Wnt family member 6; AKNA, AT-hook transcription factor; *IL27*, interleukin 27; *EBF3*, transcription factor 3; *PCNA*, proliferating cell nuclear antigen.

**Table S8.** Multiple testing corrections for prognostic effects found in the overall cohort of diffuse large B-cell lymphoma treated with R-CHOP (rituximab with cyclophosphamide, doxorubicin, vincristine, and prednisone) by the Benjamini-Hochberg method with a false discovery rate of 0.10

|  | |  | | **OS** | |  |  | **PFS** |  |
| --- | --- | --- | --- | --- | --- | --- | --- | --- | --- |
| **Factor** | | *P*-value by Log-rank test | Benjamini-Hochberg q-value | | Benjamini-Hochberg significance | | *P*-value by Log-rank test | Benjamini-Hochberg q-value | Benjamini-Hochberg significance |
| IGHV SHM^high^ | **0.011** | | 0.0231 | | **Significant** | | 0.1 | 0.12353 | Insignificant |
| IGHV SHM^high^ in *BCL2*-R^−^ | **0.006** | | 0.016275 | | **Significant** | | **0.012** | 0.036 | **Significant** |
| IGHV SHM^high^ in *MYC*-R^−^ | **0.0012** | | 0.0084 | | **Significant** | | **0.0047** | 0.0252 | **Significant** |
| IGHV SHM^high^ in MYC^−^ | **0.0058** | | 0.016275 | | **Significant** | | 0.059 | 0.09 | **Significant** |
| IGHV SHM^high^ in *BCL2*-R^+^ | 0.44 | | 0.4863 | | Insignificant | | 0.64 | 0.67 | Insignificant |
| IGHV SHM^high^ in *MYC*-R^+^ | 0.48 | | 0.504 | | Insignificant | | 0.34 | 0.3967 | Insignificant |
| IGHV SHM^high^ in MYC^+^ | 0.57 | | 0.57 | | Insignificant | | 0.67 | 0.67 | Insignificant |
| IGK/LV SHM^high^ in GCB | **<0.0001** | | 0.001 | | **Significant** | | **0.0016** | 0.021 | **Significant** |
| IGK/LV SHM^high^ in ABC | 0.24 | | 0.28 | | Insignificant | | 0.65 | 0.67 | Insignificant |
| IGHV ongoing SHM^+^ | **0.003** | | 0.01575 | | **Significant** | | 0.1 | 0.12353 | Insignificant |
| IGHV ongoing SHM^+^ in *BCL2*-R^−^ | **0.0007** | | 0.00735 | | **Significant** | | **0.01** | 0.035 | **Significant** |
| IGK/LV ongoing SHM^high^ | **0.0047** | | 0.016275 | | **Significant** | | **0.0048** | 0.0252 | **Significant** |
| IGK/LV ongoing SHM^high^ in GCB | **0.042** | | 0.06785 | | **Significant** | | **0.033** | 0.077 | **Significant** |
| HCDR3 short length | **0.0077** | | 0.01797 | | **Significant** | | **0.002** | 0.021 | **Significant** |
| HCDR3 short length in GCB | **0.0062** | | 0.16275 | | **Significant** | | **0.0091** | 0.035 | **Significant** |
| HCDR3 short length in ABC | 0.16 | | 0.19764 | | Insignificant | | 0.054 | 0.09 | **Significant** |
| K/LCDR3 short length | **0.026** | | 0.0496 | | **Significant** | | 0.094 | 0.12353 | Insignificant |
| K/LCDR3 short length in ABC | **0.031** | | 0.05425 | | **Significant** | | 0.06 | 0.09 | **Significant** |
| IGH only-D-J-resolved or unproductive V-D-J clones in GCB | 0.13 | | 0.17063 | | Insignificant | | **0.047** | 0.08973 | **Significant** |
| IGK/L unproductive VJ | 0.11 | | 0.154 | | Insignificant | | **0.024** | 0.063 | **Significant** |
| IGK/L unproductive VJ in GCB | 0.11 | | 0.154 | | Insignificant | | **0.045** | 0.08973 | **Significant** |

**Abbreviations**: OS, overall survival; PFS, progression-free survival; IGHV, immunoglobulin heavy chain variable region gene; SHM, somatic hypermutation; GCB, germinal center B-cell–like; ABC, activated B-cell–like; *BCL2*-R, *BCL2* rearrangement ; *MYC*-R, *MYC* rearrangement; IGK/LV, immunoglobulin kappa or lambda light chain variable region gene.CDR3, complementarity determining region 3.

**Note:** The order of factors listed are according to heavy chain and light chain factors but not the significance by Benjamini-Hochberg procedure. Light chain factors are in shading. Significant values are in bold.
